# Mesolimbic Dopamine Encodes Prediction Errors in a State-Dependent Manner

**DOI:** 10.1016/j.celrep.2016.03.031

**Published:** 2016-03-31

**Authors:** Georgios K. Papageorgiou, Mathieu Baudonnat, Flavia Cucca, Mark E. Walton

**Affiliations:** 1 Department of Experimental Psychology, University of Oxford, 9 South Parks Road, Oxford OX1 3UD, UK; 2 Department of Biomedical Sciences, University of Cagliari, Via Ospedale 72, 09124 Cagliari, Italy

## Abstract

Mesolimbic dopamine encodes the benefits of a course of action. However, the value of an appetitive reward depends strongly on an animal’s current state. To investigate the relationship between dopamine, value, and physiological state, we monitored sub-second dopamine release in the nucleus accumbens core while rats made choices between food and sucrose solution following selective satiation on one of these reinforcers. Dopamine signals reflected preference for the reinforcers in the new state, decreasing to the devalued reward and, after satiation on food, increasing for the valued sucrose solution. These changes were rapid and selective, with dopamine release returning to pre-satiation patterns when the animals were re-tested in a standard food-restricted state. Such rapid and selective adaptation of dopamine-associated value signals could provide an important signal to promote efficient foraging for a varied diet.

## Highlights

• Dopamine reward prediction errors are shaped by physiological state
• Both choices and dopamine signals rapidly update after selective satiation
• In a new state, dopamine signals mainly only update with experience
• When returning to a familiar state, dopamine immediately signals stored values

## Graphical Abstract

**Figure:**
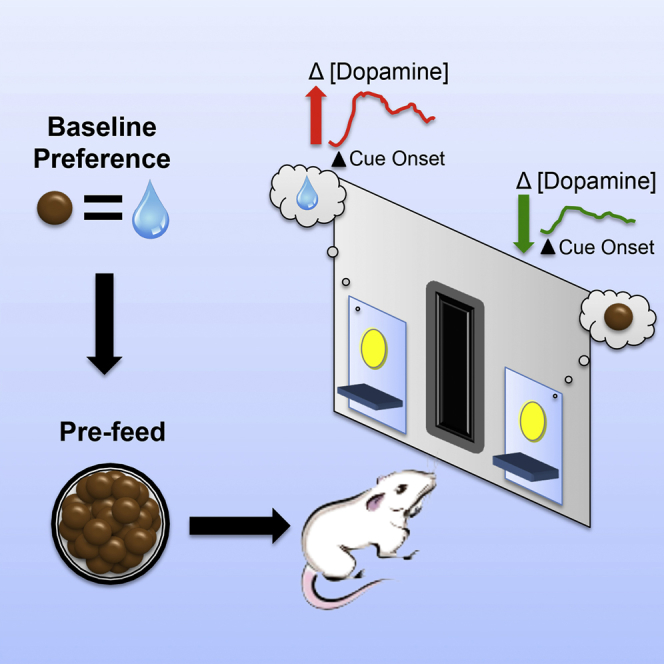

## Teaser

Dopamine signals information about reward value used for learning and decision making. Papageorgiou et al. show that mesolimbic dopamine coding of reward prediction errors rapidly updates to reflect current state-dependent values.

## Introduction

The phasic activity of midbrain dopamine neurons and dopamine release in regions such as the nucleus accumbens (NAc) signal predictions of future reward and discrepancies between such predictions and received reward (Gan et al., 2010, Montague et al., 1996, Schultz et al., 1997, Syed et al., 2016). These signals appear encoded on a common value scale, integrated across different reward attributes, that reflects individuals’ subjective preference for particular outcomes rather than the objective properties of reward (Lak et al., 2014). However, such preferences are not fixed but, instead, depend on an organism’s current nutritional needs, particularly in comparison with recent consumption. Several studies have shown that dopamine levels in the presence of reward are influenced by current physiological state, as well as the nutritional content of reinforcers (Ahn and Phillips, 1999, Bassareo and Di Chiara, 1999, Beeler et al., 2012, de Araujo et al., 2013, McCutcheon et al., 2012). Nonetheless, to date, the relationship between phasic dopamine, reward prediction errors, nutritional needs, and reward-guided choice remains poorly understood. Here, we investigated this issue by recording dopamine release while rats made choices between food and sucrose solution either in a baseline food-restricted state or after selective satiation on one of the two reinforcers (Rolls et al., 1983). Thus, by monitoring how patterns of dopamine release updated between the sessions, we could investigate how dopamine prediction errors are influenced by selective changes in subjective value and how value predictions and behavioral preferences updated with experience of the reinforcers in a new state.

## Results

### Behavioral Performance before and after Selective Satiation

**Figure 1:**
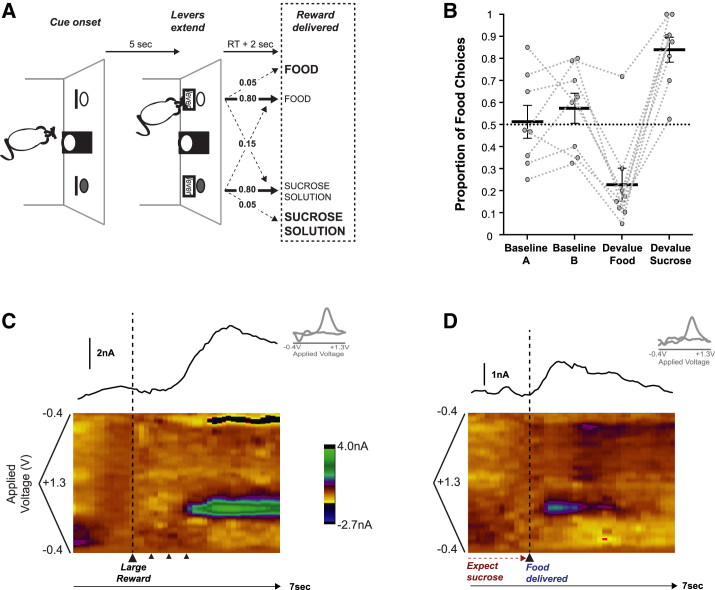
Task Design, Behavioral Performance, and Example Dopamine Signals (A) Schematic of a typical forced trial (“Forced Left”). Arrows between “Levers extend” and “Reward delivered” indicate the transition probabilities following a response on that option (“FOOD” and “SUCROSE SOLUTION” in regular type indicates standard reward; in bold type, they indicate increased reward). RT, response time. (B) Proportion of food choices on choice trials (circles correspond to individual rats). (C and D) Individual example MORE (C) and SWITCH (D) trials. Each panel depicts the recorded current × applied voltage in a pseudocolor plot from 2 s before and 5 s after reward delivery. The upper trace depicts the extract dopamine signal, along with an example cyclic voltammogram identifying the detected current as dopamine. All averages indicate mean ± SEM.

Food-restricted rats were trained to perform a two-option operant decision-making task where the selection of each option was associated with a particular type of reward (food pellet or sucrose solution) (Figure 1A). Sessions consisted of trials where only one reward type was available (“forced” trials) and others where rats could choose between the two (“choice” trials). After acquiring the task, the rats (n = 8) performed four sessions: two baseline sessions (A and B), each of which preceded a devaluation session (the devalue food session and the devalue sucrose solution session, order counterbalanced across animals) that was identical to the baseline sessions, except that the rats had free access to one of the rewards for an hour before the test session.

In the first pre-devaluation baseline session (baseline A), the group of rats overall displayed no overall preference in general for either reward type on choice trials (t test against 50% for food choices: t(7) = 0.168, p = 0.87), no difference in response latencies to the two options on forced trials (t(6) = 0.94, p = 0.38; the data from one animal was lost because of a computer error), and no differences in the numbers of wrong-lever choices or missed trials (both <2% of trials, t(7) < 1.60, p > 0.15).

Prior to the devaluation sessions, the rats consumed, on average, either 11.5 g (SEM, ±1.24 g) of pellets or 24.75 ml (SEM, ±1.81 ml) of sucrose solution. This manipulation reliably altered the animals’ preference for the reward types (t test against 50% for food choices: devalue food, t(7) = −2.84, p = 0.01; devalue sucrose solution, t(7) = 4.13, p < 0.01) (Figure 1B). There was no difference in the magnitude of this change following satiation with either the food or the sucrose solution (t(7) = 0.29, p = 0.78). There was also a significant increase in the number of missed trials and wrong-lever choices on forced trials in the devaluation sessions compared to baseline sessions (main effect of devaluation, both Fs(1, 7) > 7.02, p < 0.034), an effect driven by a selective increase on the devalued option (interaction between reward type and devaluation session: wrong choices, F(1, 7) = 9.75, p = 0.02; missed trials, F(1, 7) = 5.53, p = 0.051).

These changes in subjective valuation were temporary and specific to the devaluation session. Preference returned to indifference in the baseline B session run in between the counterbalanced devaluation sessions (t(7) = 1.08, p = 0.32), and there was no change in choices from the pre-devaluation baseline session (t(7) = 1.18, p = 0.28).

### Dopamine Release at Reward Delivery following Sensory-Specific Satiation Procedures

We monitored dopamine release in the NAc core (Figure S1) using fast-scan cyclic voltammetry while rats performed this reward identity decision paradigm. On 80% of trials, the choice of one lever resulted in the delivery of the standard amount of the expected reward type (“standard” trials). However, on the remaining subset of trials, the animals received either (1) an increased quantity of the expected reward type (value surprise “MORE” trials) or (2) the standard amount of the other reward type (identity surprise “SWITCH” trials) (Figures 1A, 1C, and 1D). Note that, until the reward is dispensed, surprise trials are otherwise identical to standard trials. While we recorded dopamine release in both baseline and selective satiety sessions, here we will mainly focus on patterns of dopamine release in the latter.

**Figure 2:**
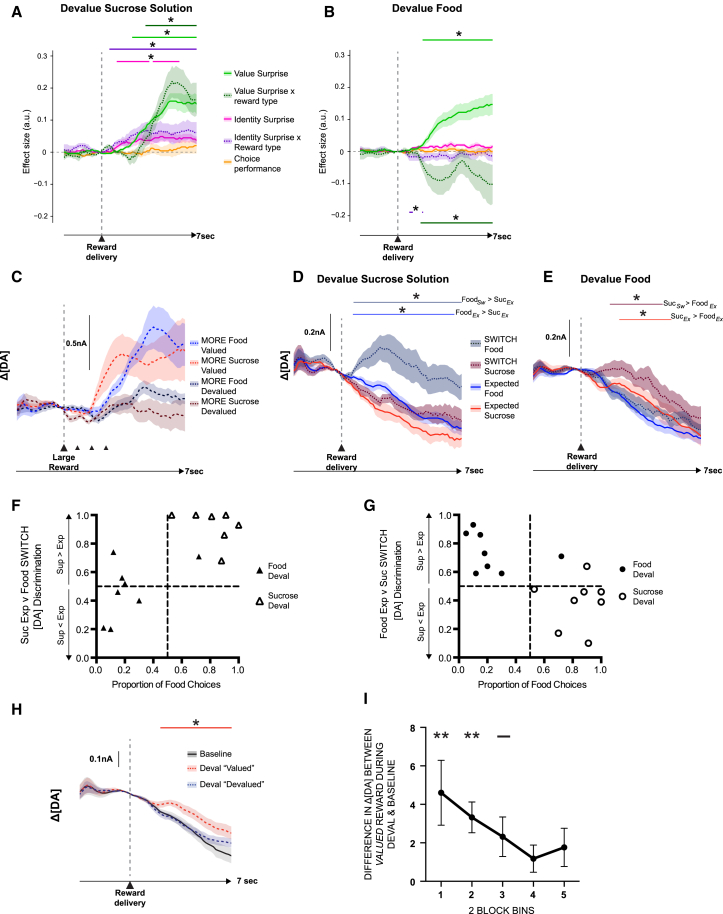
Dopamine at Reward Delivery after Selective Satiation (A and B) Average effect sizes from a general linear model of post-reward dopamine signals after sucrose solution (A) or food (B) devaluation. (C) Average dopamine release on MORE food or sucrose trials divided up by the reward type that was devalued prior to the session. (D and E) Dopamine signals on expected (Exp) and SWITCH (Sw) trials after sucrose (Suc) (D) or food (E) was selectively devalued. (F and G) Dopamine discrimination index for each animal in a 5 s post reward window for SWITCH food versus expected sucrose (F) or SWITCH sucrose versus expected food (G) plotted against each animal’s food choices. Data are separated into food (filled symbols) or sucrose solution (open symbols) devaluation sessions. (H) Comparison of dopamine signals when receiving expected reward in baseline and devaluation sessions as a function of which reward type was devalued. (I) Difference between average dopamine release after reward delivery when receiving the valued option in devaluation sessions and this same reward type in the previous baseline session (collapsed over reinforcers), divided into five bins each of two blocks of trials. Lines: ^∗^p < 0.05 permutation tests, corrected for multiple comparisons; ^∗∗^p < 0.05; ^−^ p = 0.058, two-tailed t test against 0. All averages indicate mean ± SEM. DA, dopamine.

To examine the effect of selective satiety and, consequently, a selective change in the subjective value of one of the options, on value-related dopamine signals at the time of reward delivery, we ran a linear regression on the two devaluation sessions (Figures 2A and 2B). There was a strong influence of MORE trials on dopamine release, as well as a significant interaction between MORE trials and reward type. Importantly, the sign of the interaction term switched depending on whether food or sucrose solution was devalued (Figure 2C). This demonstrates reinforcer-specific satiety effects on value surprise trials. The same influence of selective satiety was also observed on SWITCH trials. In sucrose solution devaluation sessions, there was a transient increase in dopamine following the surprise delivery of a valued food pellet after a response on the sucrose solution lever (Figures 2A, 2D, 2F, and 2G). These signals were significantly more discriminable than during the baseline session (paired t test on the dopamine discrimination index: t(7) = 2.88, p = 0.028). The opposite pattern was observed in the food devaluation sessions: now, it was the surprising delivery of the valued sucrose solution that caused a selective increase in dopamine release, whereas there was no observable increase following a surprise pellet delivery (Figures 2B and 2E–2G). Therefore, surprise-evoked dopamine release can also be modulated by the current state-based value of the reinforcers, demonstrating that the pattern of dopamine is distinct from the physical properties of the reward.

It was also evident that dopamine release differed even on the standard trials for the valued and devalued options in a new state, even though the anticipated type of reward was always delivered (p < 0.05; Figures 2D and 2E). To investigate what might be influencing this, we directly contrasted dopamine time locked to reward delivery in the devaluation sessions against an equivalent period recorded in the baseline session (Figure 2H). Surprisingly, there was no consistent difference in the change in average dopamine levels after receipt of the *devalued* reward when compared to receiving that same reward in the baseline session (p > 0.05). Instead, there was a small but significant increase in dopamine when receiving the *valued* option for both reinforcers when compared to the same situation during baseline testing (Figure 2H; Figure S2). Moreover, when the session was divided into five blocks, this difference was found to be, on average, largest at the beginning of the session and diminished linearly as the session progressed (linear main effect of block: F(1, 7) = 5.88, p = 0.046) (Figure 2I). Therefore, receipt of the valued reward following selective satiety procedures appeared to produce a small positive prediction error that updated as the animals gained more experience of the reward in the new state.

### Rapid Updating of Cue-Elicited Dopamine Release after Changes in State

The pattern of dopamine release at reward delivery suggests that value predictions are shaped by current motivational state in a reinforcer-specific manner. If so, state-based modulations of value predictions should also be observable at cue onset.

**Figure 3:**
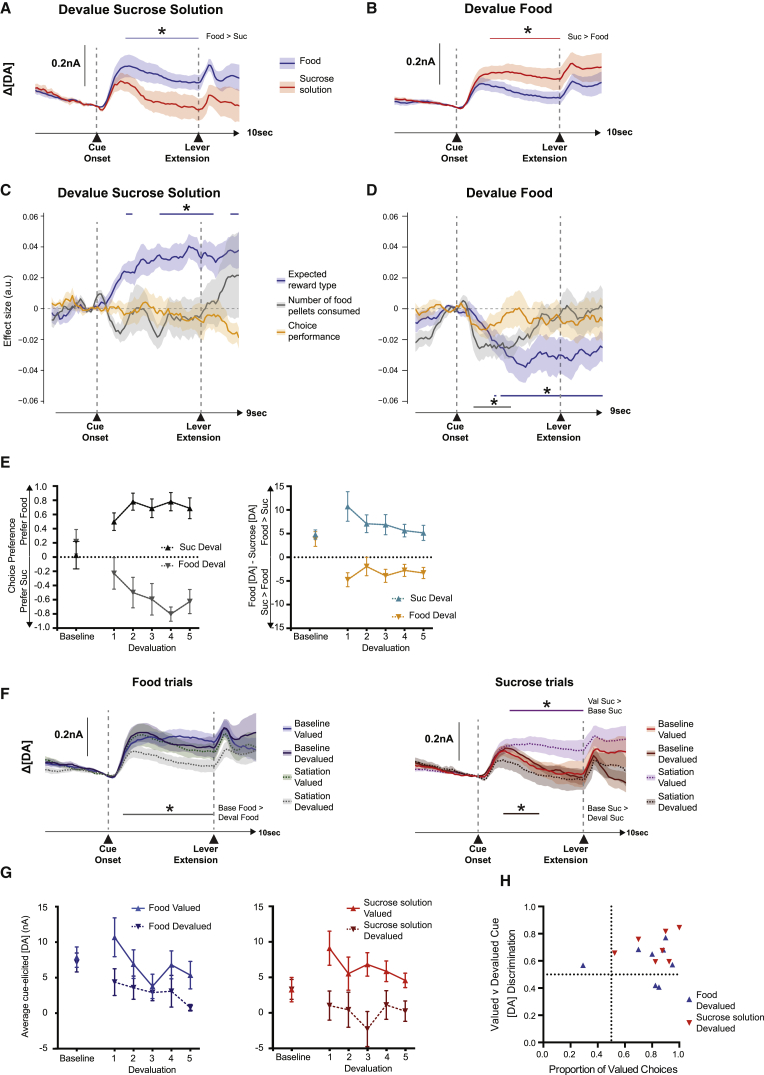
Dynamic Changes in Cue-Evoked Dopamine after Selective Satiation (A–D) Average cue-evoked dopamine (DA) signals (A and B) or effect sizes from a general linear model (C and D) after selective satiation on sucrose solution (Suc) (A and C) or food (B and D). (E) Change in preference and relative cue-evoked dopamine plotted over five bins each of two blocks of trials following sucrose solution or food devaluation. Choice is expressed as a change from 50% [(2 × proportion of food choices) − 1]. Relative cue-evoked dopamine is the difference between average food cue and sucrose solution cue dopamine levels during the 5-s post-cue period. The average difference across the whole of the immediately preceding baseline session is presented for comparison. (F) Comparison between average cue-evoked dopamine release during the baseline and devaluation sessions on food (left) or sucrose solution trials (right). Data are divided up into “valued” and “devalued” based on which reinforcer the rats had free access to before the devaluation session. Baseline data are from the immediately preceding baseline session. (G) Average dopamine in the 5-s post-cue period in the devaluation sessions, divided up into five bins each of two blocks of trials. The average difference across the whole of the immediately preceding baseline session is presented for comparison. (H) Dopamine discrimination index for each animal in the 5 s post cue period for both valued food versus devalued sucrose solution (red triangles) and for valued sucrose solution versus devalued food (blue triangles), plotted against each animal’s choices of the valued reinforcer in that session. There was no reliable relationship between these measures (r = 0.183, p = 0.51). Lines: ^∗^p < 0.05 permutation tests, corrected for multiple comparisons. All averages indicate mean ± SEM.

As can be observed in Figure 3, this is exactly what we found. While food cues elicited significantly greater dopamine release than sucrose solution cues in the sucrose solution devaluation session (Figure 3A), this reversed after food devaluation, with dopamine release for food cues now lower than after sucrose solution cues (Figure 3B). This was borne out by a linear regression that showed a significant effect of reward type on cue-evoked dopamine in both sessions, but with the sign modulated by the identity of the pre-fed reinforcer (Figures 3C and 3D). To further investigate this change in cue-elicited release, dopamine levels either on forced food or forced sucrose solution trials were analyzed across five equally sized blocks in the session. This showed that these effects occurred rapidly, being evident within the first block of the session (analysis of valued or devalued cue dopamine: F(1, 6) = 11.93, p = 0.014; no main effect or interaction with reward type: both Fs < 1.39, p > 0.28; n = 7, as one animal was excluded for having ≤5% responses on the devalued option) (Figure 3E).

Interestingly, although selective satiation uniformly decreased dopamine after *devalued* cues across the session, there was an asymmetric effect on *valued* cue-elicited dopamine (Figures 3F and 3G). Specifically, after food devaluation, dopamine levels were, on average, significantly greater after sucrose solution cues compared to baseline sessions (p < 0.05). By contrast, there was no statistically reliable change in either direction in response to valued food cues after sucrose solution devaluation. In other words, after eating to satiety, the predicted value of the sweet liquid option increased. Nonetheless, while the selective satiety procedures reliably biased choice behavior and modulated dopamine release, there was no observable consistent relationship between the size of cue-elicited signals in a particular session for a particular animal and its preference for one reinforcer over the other on choice trials (Figures 3C–3E and 3H).

**Figure 4:**
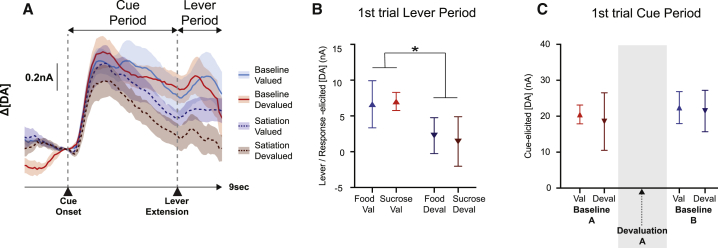
Cue-Elicited Dopamine Release on First Trials of the Session (A) Average dopamine levels in a 5-s window after cue onset on the first food/sucrose solution trial in baselines A and B. Baseline data are divided into “valued” (Val) or “devalued” (Deval) based on which reinforcer the animals had free access to in devaluation A. (B) Average dopamine signals after cue onset on the first food/sucrose solution trial in the baseline and devaluation sessions. Baseline data here are divided up based on which reinforcer the animals had free access to in the subsequent devaluation session. (C) Average dopamine levels in a 2-s post-lever extension window (prior to reward delivery) on the first food or sucrose solution trial, averaged across the devaluation sessions. Levels were significantly reduced on the first devalued trial compared to the first valued trial (^∗^p < 0.05, ANOVA). All averages indicate mean ± SEM. DA, dopamine.

Although these analyses show a rapid influence of selective satiety on dopamine release, it is not clear whether this is purely an experience-dependent effect based on learning the value of the options in the new state or whether dopamine cue signals can update even before the devalued reward is consumed during the session. To examine this, we analyzed dopamine release elicited by the first presentation in the session of both the valued and devalued options (Figure 4A). This revealed an overall attenuation of dopamine release on the initial trial of the devaluation sessions compared to the preceding baseline session (main effect of session type: F(1, 6) = 12.44, p = 0.012, including trial order as a between-subjects factors). However, this reduction was not significantly greater after first presentation of the cue associated with the currently devalued option than after that associated with the currently valued option (interactions including Session Type × Devaluation: all Fs < 0.27, p > 0.62). This implies that selective satiety induces an immediate general, rather than stimulus-specific, reduction in cue-elicited dopamine signals but that rats need experience of the reinforcer in the new state to fully update learned cue associations.

Nonetheless, inspection of Figure 4A suggests that dopamine levels were, on average, lower on the first devalued trial compared to cue presentations in valued states, particularly in the later period between lever extension and reward delivery. Therefore, we also directly compared average dopamine levels during the lever extension/response period, prior to reward delivery, on the first valued and devalued trials. This confirmed that dopamine levels were significantly attenuated after presentation of the devalued lever, compared to after the valued lever, even though the reinforcers had yet to be directly experienced in the new state (main effect of devaluation: F(1, 6) = 12.63, p = 0.012) (Figure 4B). This occurred in spite of the fact that there were no differences in lever press latency between the first valued or devalued trial (mean ± SEM: valued, 0.42 s ± 0.09 s; devalued, 0.60 s ± 0.20 s; t(7) = 1.05, p = 0.33).

Importantly, although selective satiation strongly modulated dopamine levels in the devaluation session, this did not have a lasting influence over patterns of dopamine release. The first presentation of the previously devalued option in baseline B immediately elicited comparable levels of dopamine as when that same cue had been presented during the first baseline A session (comparison between first trial dopamine in baseline A and baseline B, separated by which option was devalued during devaluation A: all Fs < 0.96, p > 0.36) (Figure 4C). Therefore, while dopamine signals update with experience of the reinforcer in the new state, they immediately revert to the original learned values once animals return to a baseline food-restricted state.

## Discussion

These results demonstrate that mesolimbic dopamine flexibly encodes reward prediction error signals shaped by the specific properties of a reward to satisfy a current need. Midbrain dopamine neurons in primates tested for multiple days in a similar state have been shown to encode reward prediction errors that reflect the animals’ subjective preference for different reward types (Lak et al., 2014). Here, we observed a rapid, experience-driven updating of NAc core dopamine signals, both to predictive cues and reward delivery, to reflect the subjective value of stimuli following selective satiation.

Several of our results, therefore, appear consistent with key predictions of model-free temporal difference learning models. Dopamine release on SWITCH trials in the devaluation sessions principally encoded surprising changes in reward identity based on discrepancies between expected and received value rather than the sensory surprise of receiving the alternative reinforcer. This does not rule out that coding of reward identity prediction errors may exist in other contexts, where the value difference between the options is less prominent or when a change in identity is more relevant for behavior. For instance, the SWITCH trials here occurred as rare fluctuations in an otherwise stable task, but in other paradigms, such as unblocking or reversal learning, a change in reward identity can be more long lasting and of more significance for behavior (McDannald et al., 2014, Stalnaker et al., 2014). Equally, it is possible that distinct dopamine pathways might contain additional information about reward identity or other aspects of reward (Huetteroth et al., 2015). The current data were collected from the NAc core, as dopamine release in this structure has been shown to signal discrepancies from expectation (Day et al., 2007, Syed et al., 2016). However, in rodents, the NAc shell rather than the core—and, specifically, the D1-receptor-expressing medium spiny neurons in this region—has been associated with the ability of specific reinforcers to motivate and invigorate responding and promote feeding (Corbit and Balleine, 2011, Laurent et al., 2014, O’Connor et al., 2015).

We observed a strong modulation of both food- and sucrose-solution-elicited dopamine by the amount of reinforcer consumed within and prior to the session. Such selective modulation of stored value signals by specific satiety may be important to promote efficient and varied foraging behaviors. Cue-elicited dopamine release rapidly updated, with significant differences between the valued and devalued signals being evident within the first block of trials following selective satiation. However, this appeared to be predominantly shaped by direct incentive learning in the new state. While there was a general reduction in dopamine on the first trials of the devaluation sessions, compared to the preceding baseline sessions, this was not selective for the devalued option. This is in line with studies showing a general activating role for the NAc core, and dopamine transmission in this region, in the presence of reward-associated cues to motivate and invigorate available actions (Corbit and Balleine, 2011, du Hoffmann and Nicola, 2014).

However, it is notable that there were already selective differences in dopamine levels in the period after lever extension while the rat was making a response and waiting for either the valued or devalued reward. Therefore, some aspects of dopamine signaling can partially update without direct experience of the outcome (Bromberg-Martin et al., 2010). Moreover, cue-elicited dopamine returned to pre-devaluation patterns by the start of the subsequent baseline session run in a food-restricted state, in spite of the large difference between release elicited by the valued and devalued cues at the end of the devaluation session. This implies that mesolimbic dopamine systems have access to stored memories of learned incentive values when returning to a familiar state.

Together, our data add to the evidence indicating a close link between mesolimbic dopamine and physiological state (de Araujo et al., 2012, McCutcheon, 2015, Sclafani et al., 2011). Ventral tegmental area dopamine neurons receive excitatory inputs from the lateral hypothalamus (Watabe-Uchida et al., 2012) and dopamine cell activity, and NAc core dopamine release is influenced by physiological state (Branch et al., 2013) and by peptides involved in appetite (Cone et al., 2014). In the present experiment, decisions will be made based not only on the objective sensory qualities of a food pellet versus a bolus of sucrose solution but also on their subjective value in a given state. The rapid adaptation of mesolimbic dopamine signals following a change in state would potentially allow it to play an important role in prioritizing behaviors based on the available opportunities.

## Experimental Procedures

### Animals

17 male Sprague-Dawley rats were used for this experiment, of which 8 contributed data reported here (see the Supplemental Experimental Procedures). During the training and testing periods, access to food was restricted so that rats’ weights were kept between 85% and 90% of their free-feeding body weight. Water was continuously available in the home cages. All procedures were in compliance with the United Kingdom Animals Scientific Procedures Act of 1986 and the University of Oxford Policy on the Use of Animals in Scientific Research. All experiments were approved by the University of Oxford Animal Welfare and Ethical Review Board.

### Behavioral Paradigm

We used fast-scan cyclic voltammetry to record dopamine release from chronically implanted carbon fiber electrodes in the NAc, as described previously (Clark et al., 2010, Syed et al., 2016), as animals performed a two-option/two-reward decision-making task. Sessions consisted of 120 trials, broken down into blocks of eight forced trials (four to each lever in a pseudorandom order) followed by four free-choice trials. One option was consistently associated with one reward type (45-mg food pellet), and the other was associated with a bolus of sucrose liquid (95 μl 20% sucrose solution), both delivered to the same food cup. On 80% of trials, animals received the reinforcer associated with the selected option. However, on 10% of the forced trials and 5% of the choice trials, the animals unexpectedly received the reward associated with the other lever (“SWITCH”). On another 5% of the forced trials, the animals received four times more reward than expected, although of the expected identity (“MORE”). These surprise trials occurred pseudorandomly throughout the session.

### Data Analysis

As in previous studies, dopamine signals were extracted using principal-component analysis (Heien et al., 2004, Syed et al., 2016). To quantify which factors affected dopamine levels, regression coefficients were estimated for each animal at each time point around an event of interest. A linear model was used with a constant term, representing an ordinary least-squares fit of the given regressors to the data over trials (see the Supplemental Experimental Procedures). The discriminability of dopamine signals in pairs of different trial types was analyzed in each individual animal at each time point using the area under the receiver operating characteristic curve (auROC) (Syed et al., 2016). All data are reported as significant based on permutation tests when p < 0.05, corrected for multiple comparisons (i.e., p < 0.001). To calculate a dopamine discriminability index, we calculated the auROC using the average dopamine in the 5-s window after reward delivery for a particular trial type. In situations where there were insufficient numbers of trials to calculate an auROC (i.e., when examining changes in bins of trials across the session), we extracted the average dopamine levels instead, within a 3-s window after reward delivery, a 5-s window between cue onset and lever extension, or a 2-s window between lever extension and reward delivery, and performed a repeated-measures ANOVA.

## Author Contributions

G.K.P. and M.E.W. conceived the study; G.K.P. and M.B. performed surgeries; G.K.P. collected the data with the assistance of F.C.; M.E.W. and G.K.P. analyzed the data; and M.E.W. and G.K.P. wrote the manuscript.

## Supplemental Information

Document S1. Supplemental Experimental Procedures and Figures S1 and S2

Document S2. Article plus Supplemental Information
