# Supplementary material for: Mesolimbic Dopamine Encodes Prediction Errors in a State-Dependent Manner
Source: Cell Rep. 2016 Mar 31;15(2):221–8. doi: 10.1016/j.celrep.2016.03.031 (PMC4835543; doi:10.1016/j.celrep.2016.03.031)
Supplement: Document S1. Supplemental Experimental Procedures and Figures S1 and S2 [file mmc1.pdf]

Cell Reports, Volume *15*

Supplemental Information

Mesolimbic Dopamine Encodes Prediction Errors in a State-Dependent Manner

Georgios K. Papageorgiou, Mathieu Baudonnat, Flavia Cucca, and Mark E. Walton

Supplementary Experimental Procedures

Animals

17 male Sprague-Dawley rats weighing 350-400g were used for this experiment (Harlan Olac, Bicester, UK). This included 11 naïve rats aged 3-6 months at the start of testing as well as an additional 6 rats, aged 6 months, which had previously participated in a separate 2-option appetitive decision making experiment. A total of 6 rats were excluded from the electrochemistry analysis, 3 for misplaced electrodes outside the nucleus accumbens core and 3 for broken/noisy electrodes. In addition, 3 rats did not perform the Devaluation sessions and so are not included in the analyses here, leaving a total of 8 rats. As we wanted to relate dopamine release to behaviour, we have only included data from 1 electrode in each individual. Animals were maintained on a 12-hr light/dark cycle, were initially grouped housed in cages of 3 but then were individually housed after surgery and during the testing period. All testing was carried out during the light phase. During the training and testing periods, access to food was restricted such that rats' weights were kept between 85-90% of their free-feeding body weight. Water was continuously available in the home cages. All procedures were in compliance with the United Kingdom Animals Scientific Procedures Act (1986).

Surgical Procedures

Animals were anaesthetised using isoflurane (4% induction and 1.5% for maintenance) and given buprenorphine (Vetergesic, 0.1 ml/kg) at the start of the surgical procedure. Body temperature was maintained at 37±0.5 C with the use of a homeothermic heating blanket. Corneal dehydration was prevented with application

of ophthalmic ointment (Lacri-Lube, Allergan, UK). After induction, the rat's head was shaved and secured in a stereotaxic frame. The head was then cleaned with dilute hibiscrub, 70% alcohol and a local anaesthetic, bupivacaine, was applied to the area. The scull was then exposed and holes were drilled for an Ag/AgCl reference electrode (AP: -3.7, ML: -1.4), 4 anchoring screws (Precision Technology Supplied Ltd, UK) and a recording electrode in each hemisphere. After the screws were secured and the reference electrode inserted, custom-made carbon fibre microelectrodes were then lowered into the NAc core (AP: +1.4, ML: ±1.3, DV: 7.0). The carbon fibre microelectrodes and reference electrode were attached to a headstage connector, which was secured in place along with an anchoring headpost using dental cement (Kemdent, Swindon, UK). Following surgery, animals were administered additional buprenorphine (0.1 ml/kg) and meloxicam (Metacam, 0.2 ml/kg). Meloxicam was also administered for at least 3 days following surgery. Animals had on average three weeks of post-surgery recovery with food and water *ad libitum*, prior to food restriction and further behavioural training/recording.

Behavioural Paradigm

Apparatus

Testing was carried out in custom-designed operant chambers (30.5 x 24.1 x 29.2 cm; Med Associates, VT, USA). Each chamber was housed within a sound-attenuating cabinet ventilated with a fan, which provided constant background noise of ~64dB. Each chamber contained two retractable levers, situated 9.5cm on either side of a reward magazine that contained a receptacle into which both 45mg standard grain-based pellets (Test Diet, distributed through Sandown Scientific, UK)

and sucrose solution could be delivered. Above each lever there was a cue light. The magazine was fitted with an infrared beam that signalled when animals entered the receptacle. Each chamber was also fitted with a house-light.

Training

Rats were first given experience of each type of reward in the operant recording chamber by placing a number of pellets or amount of sucrose solution in the reward receptacle during two brief sessions separated by ~1 hour (reward order counterbalanced across animals). On the next day, the rats received a magazine training session with both reinforcers. In this session (duration ~30mins), pellets or sucrose solution were delivered on VI60 schedule. Subsequently, rats were taught to press levers to gain reward. Half of the rats were trained that the left lever would lead to sucrose solution and the right lever to pellets, whereas the other half were trained on the opposite configuration. These associations remained fixed throughout the data acquisition. Once the animals were making ~100 responses on both levers, they moved on to the main paradigm.

The main paradigm, used during both behavioural and subsequent voltammetric recording sessions, consisted of individual trials where animals could press a lever to gain one of the two reward types. Sessions could contain 'forced' trials where only one reward type was available, and 'free choice' trials where both reward types were available. At the start of a forced trial, the house light would turn on and one of the two cue lights would illuminate. After a delay of 5s, both levers would extend. A single lever press on the option under the illuminated cue would cause both levers to retract and reward to be delivered to the receptacle 2s later.

The cue light over the selected option remained illuminated during this delay to reduce any working memory load between choice and reward delivery. There was then an inter-trial interval (ITI) of 25 – 35s. A response on the lever under the non-illuminated cue light was counted as an error and did not lead to any reward delivery. If the rat failed to make a response within 10s, this trial was counted as an omission. After either an error or omission, the levers would retract, cue and house lights turn off, and the ITI would immediately start. Choice trials were identical to forced trials except that both cue lights would illuminate and selection of either option within 10s would result in reward delivery 2s after this choice. The houselight was on throughout the session.

Reward value titration

To establish the association between each response option and a specific reward during titration sessions, the rats were first given a session with only forced trials (40 to each lever in a pseudorandom order). They then had a session where forced trials were interspersed with equal numbers of choice trials, which could be used to determine each animal's preference for the reward types. Each session consisted on 80 trials made up of blocks of 4 forced trials (2 to the left, 2 to the right lever, pseudorandom order) followed by 4 choice trials. Based on pilot data in separate animals, rats were tested with 95µl of 20% sucrose solution across two separate sessions. Our criteria for determining whether this was an appropriate volume and concentration of sucrose for the group of rats were (a) stable performance across two Pre-Test titration sessions (no significant change across the sessions) and (b) average number of food choices not being significantly different from 50%.

Reward identity task

We used fast-scan cyclic voltammetry to record dopamine release as animals performed a modified version of the 2-choice/2-reward decision-making task described above. All sessions consisted of 120 trials, consisting of 10 blocks of 12 trials (8 forced trials, 4 to each lever in a pseudorandom order, followed by 4 free choice trials). On 80% of trials, animals received the reinforcer associated with the selected option ("expected reward"). However, on 10% of the forced trials and 5% of the choice trials, the animals received the reward associated with the other lever ("SWITCH" trials). On another 5% of the forced trials the animals received four times more reward than expected though of the expected identity ("MORE" trials).

There were 4 separate recording sessions, consisting of 2 baseline sessions and 2 selective satiation sessions. Selective satiation sessions had exactly the same structure with the baseline sessions but with the difference that prior to those sessions animals were given 1 hour's free access to either sucrose solution or food pellets in the testing chamber before the task commenced. During food devaluation, the animals did not have access to any fluids. The session order was always Baseline A – Selective Satiation A – Baseline B – Selective Satiation B, with the order of satiation (food or sucrose solution) counterbalanced across animals.

Fast-scan cyclic voltammetry

Fast-scan cyclic voltammetry (FSCV) recordings were made from chronically-implanted carbon fibre electrodes. Voltammetric scans were performed at a frequency of 10Hz throughout the session. Prior to a scan, the carbon fibre was held

at a potential of -0.4V (vs AG/AgCl) and then, during the scan, ramped up to +1.3V and back to -0.4V at 400 V/sec. The application of this waveform causes redox reactions in electrochemically active species, such as dopamine, at the surface of the carbon fibre that can be recorded as changes in current over time. Based on previously established criteria the recorded current in response to uncued pellet and sucrose delivery, obtained at the start and end of each recording session, was used to determine the chemical sensitivity of the recording electrode to dopamine on that particular session. An extracted cyclic voltammogram was linearly regressed against a dopamine standard, with $r^2 \geq .75$ set as the criterion based on the discriminability of dopamine from other common neurochemicals in a flow cell (Gan et al., 2010).

Data analysis

Each animal's preference for food over sucrose solution was calculated as the number of food choices / (number of food choices + number of sucrose choices). Subjective preference in each session was tested as a 2-tailed t-test against indifference (50%) and consistency across sessions as a repeated measures ANOVA with session as a within-subjects factor. Response latencies were calculated as the time from lever extension to a response.

Voltammetric analysis was initially carried out using software written in LabVIEW (National Instruments). Data were low-pass filtered at 2kHz. To isolate changes in dopamine concentration from other electrochemical signals, a principal component analysis was performed using a standard training set of stimulated dopamine release detected by chronically implanted electrodes, with dopamine treated as the first principal component among other unrelated electrochemical

fluctuations such as changes in pH (Heien et al., 2004). The data were smoothed using a 0.5s moving window. Trials where the PCA failed to successfully extract dopamine current on >50% of data points in a trial were excluded. Once dopamine-related current changes were extracted all further analysis was undertaken using Matlab® (Mathworks, MA, USA).

To quantify which factors affected dopamine levels, regression coefficients were estimated for each animal at each time point in either a 7s window spanning from 2s before reward delivery to 5s after reward or a 9s window from 2s before cue onset to 7s after (cue and lever extension periods). A linear model was used with a constant term, representing an ordinary least-squares fit of the given regressors to the data over trials.

For analysis of the reward-evoked dopamine on all correct trials, the regressors of interest were: (1) SWITCH trial, (2) MORE trial, (3) cumulative number of food pellets consumed, and (4) preference for food in the choice trials at the end of each block (transformed to be 1 for 100% preference for food and –1 for 100% preference for sucrose solution), as well as interaction terms for (5) SWITCH x reward type, (6) MORE x reward type, and (7) preference x reward type. We also included two regressors for reward type (food trials were assigned 1, sucrose solution were assigned –1) and trial type (forced 1, choice –1), though these are not presented to the sake of clarity. For analysis of cue-evoked dopamine on all correct trials, the regressors were again: (1) reward type, (2) trial type, (3) amount of food consumed, and (4) block-by-block food preference, and 3 terms for the interaction between reward type and the other regressors.

Each trial in each regressor was modeled with a single value. All regressors except for choice performance, whether continuous or categorical, were mean-centered. Regression coefficients in each animal were averaged. We focused on the significance of the regression coefficients in the 5s post-reward period or 5s post-cue onset period was tested against a population of 1000 coefficients obtained by randomly permuting the pairings between the regressors and the data (we also ran the post-cue GLM to include the 2s period after lever extension, though these data are not discussed here and statistical analyses were not adjusted to include this period). Permutation tests were considered significant at any time point when the regression coefficient from the real data exceeded the maximum or minimum of the permuted population of coefficients ($p < 0.05$, corrected for multiple comparisons over the 5s after event onset; i.e., $p < 0.001$ uncorrected).

The discriminability of dopamine signals between pairs of trial types (e.g., expected sucrose versus surprise food) was analysed in each individual animal at each time point in a 5s period after either reward delivery or cue onset (between cue onset and lever extension) using the area under the receiver operating characteristic curve (auROC). The auROC from each animal was then averaged and significant discriminability at each time point was determined using 1000 random permutations of the trial types and re-computing the auROC to generate a null distribution. Permutation tests were considered significant at any time point when $p < 0.05$, corrected for multiple comparisons (i.e., $p < 0.001$ across 50 timepoints).

For the Devaluation sessions, we combined the data across the counterbalanced food / sucrose devaluation sessions to look at the effects in the whole group for each manipulation. We here focused on (a) surprise signals

(SWITCH / MORE), but also (b) contrasted dopamine signals on the expected reward trials in sessions depending on whether the reward type was devalued or not (non-devalued is termed “valued” throughout) (e.g., expected valued sucrose versus expected devalued food / expected valued food versus expected devalued sucrose). As there were only a few value surprise trials in each session, it was not possible to directly compare these trial types using the auROC approach. Therefore, instead, we found the average signal in a 3s window, from 0.5-3.5s after reward delivery, and compared these using a repeated measures ANOVA with within subjects factors devaluation session and reward type and devaluation order as a between subjects factor (NB. the results were unchanged if a peak measure was taken instead). To examine changes in dopamine release after a state change on standard trials, we combined the data across reward types in the Selective Satiation sessions into valued and devalued trials and then, using an auROC, compared dopamine on expected reward dopamine on these two trial types against signals recorded in the baseline session. We also did comparable analyses on the data from each reinforcer in isolation. To look at how the valued signals changed during the session, we first broke these sessions down into 5 bins, each containing 2 blocks of trials (i.e., 8 forced food and 8 forced sucrose solution) and subtracted the average dopamine signal in a 0.5-3.5s window after reward delivery on expected reward trials in the Baseline session and the valued expected reward trials during the Devaluation session. To account for variance in the data, we log transformed the values before running a repeated measures ANOVA, with trial bin as a within-subjects factor, and *post-hoc* 1-sample t-tests against zero to determine in which bins there was significantly greater dopamine during the valued trials than baseline trials.

We also analysed the cue data from the selective satiation sessions in a similar manner. First, we again compared all forced trials (food versus sucrose solution) either in food devaluation or sucrose solution devaluation sessions. We then examined dopamine release following presentation of food or sucrose solution cues in a valued or devalued state and contrasted it with dopamine release to that cue in the immediately preceding Baseline session (i.e., if food was devalued in Selective Satiation session B for a particular rat, the Baseline B data was used for comparison whereas if it was devalued in Selective Satiation session A, Baseline A data was used). Once again, the average dopamine levels in the 5s post-cue period on forced trials were taken for each of 5 equally sized bins and analysed. In particular, we focused on changes observed in the first 2-block bin (1st 8 forced trials for each reinforcer).

Finally, we examined the cue data just from the first food and sucrose solution trial in each session before the animals have experienced each reinforcer in the session. If the animal made an error or did not respond on one of these trials, we instead took the first trial where a response was correctly made. We not only extracted the average dopamine signals in a 5s window after cue onset but also in a 2s window after lever extension, during which animals made a lever press response. Note that as there was a 2s delay between the response and reward delivery, none of these signals were contaminated by post-reward changes in dopamine.

Histology

At the end of the experiment, the rats were deeply anaesthetized with sodium pentobarbitone (200mg/kg) and electrolytic microlesions were made at the

electrode locations before they transcardially perfused with saline followed by a 10% formol saline solution. Brains were extracted and placed into a formol saline solution. Subsequently, the brains were placed in a sucrose/formalin solution for 24 hours, before being frozen and sectioned in 50 µm coronal slices using a cryostat. Sections were stained with cresyl violet and electrode locations were verified by two experimenters to confirm the electrode locations (Fig.S1).

Supplementary Figures

mm from Bregma

+ 2.52

+ 2.16

+1.8

+1.68

+1.08

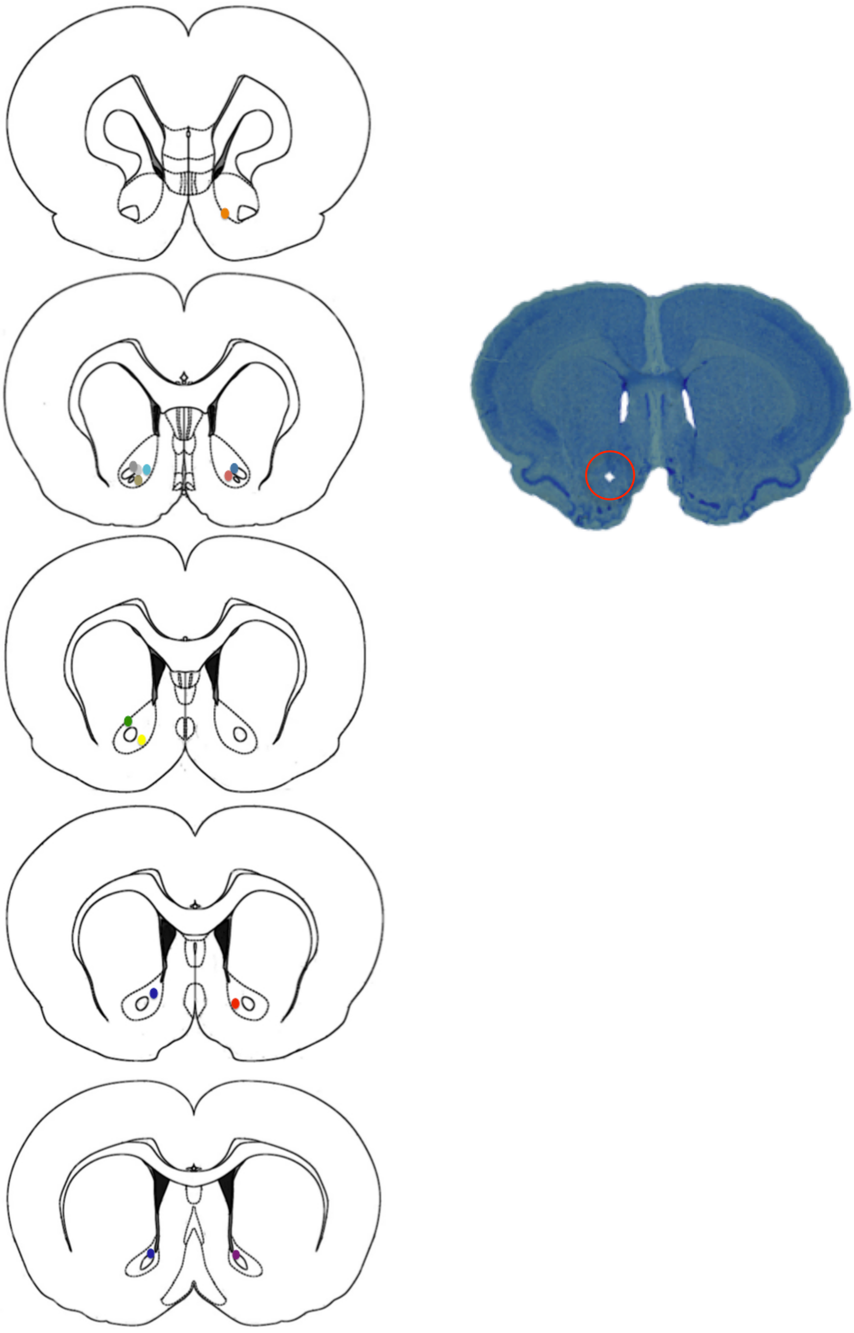

Figure S1. Representation of Recording Sites, Related to the FCV Data in the Results. Schematic, along with an example photomicrograph, of the recording locations in the nucleus accumbens core. The blue dot on the schematic shows the location of the electrode indicated by the red circle on the photomicrograph. The numbers next to each section indicate distance in mm anterior to bregma. Adapted from the atlas of Paxinos and Watson (2005).

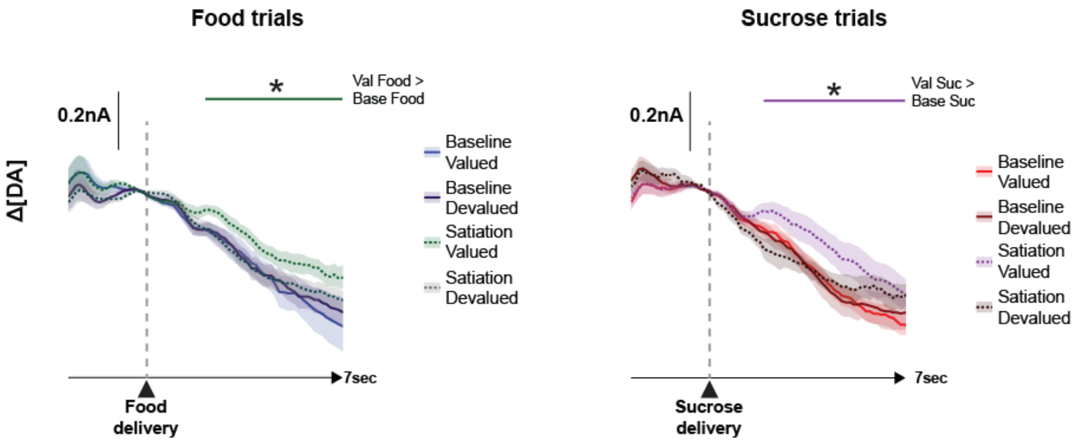

Figure S2. Comparison Between Dopamine Release in Baseline and Devaluation Sessions, Related to Figure 2. Dopamine release time-locked to reward delivery for expected food (left panel) or sucrose solution (right panel) in either the Baseline or Devaluation session. The data are divided up based on which reinforcer type rats had free access to before the Devaluation session: the represented reward ("Satiation Devalued") or the other reward ("Satiation Valued"). Baseline data are from the immediately preceding Baseline session. Therefore, if Food was devalued in Session 2 and Sucrose Solution in Session 4, Baseline A data would be "Baseline Devalued" or Baseline B data would be "Baseline Valued" for Food trials and vice versa for Sucrose Solution trials.
