# Supplementary material for: Mesolimbic Dopamine Encodes Prediction Errors in a State-Dependent Manner
Source: Cell Rep. 2016 Mar 31;15(2):221–8. doi: 10.1016/j.celrep.2016.03.031 (PMC4835543; doi:10.1016/j.celrep.2016.03.031)
Supplement: Document S2. Article plus Supplemental Information [file mmc2.pdf]

Cell Reports

Report

Mesolimbic Dopamine Encodes Prediction Errors in a State-Dependent Manner

Graphical Abstract

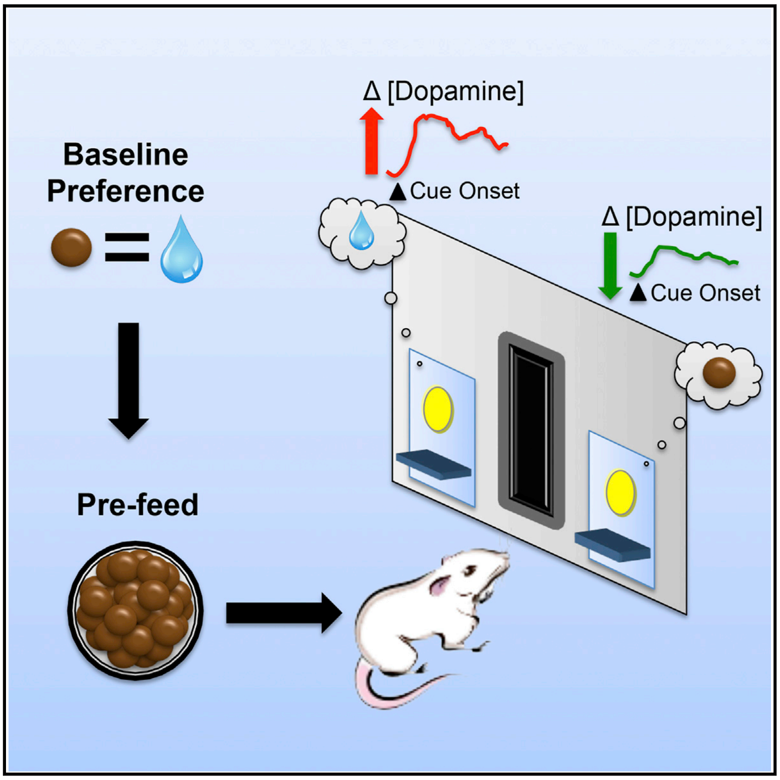

Highlights

- Dopamine reward prediction errors are shaped by physiological state
- Both choices and dopamine signals rapidly update after selective satiation
- In a new state, dopamine signals mainly only update with experience
- When returning to a familiar state, dopamine immediately signals stored values

Authors

Georgios K. Papageorgiou, Mathieu Baudonnat, Flavia Cucca, Mark E. Walton

Correspondence

georgios.papageorgiou@psy.ox.ac.uk (G.K.P.), mark.walton@psy.ox.ac.uk (M.E.W.)

In Brief

Dopamine signals information about reward value used for learning and decision making. Papageorgiou et al. show that mesolimbic dopamine coding of reward prediction errors rapidly updates to reflect current state-dependent values.

CrossMark

Papageorgiou et al., 2016, Cell Reports *15*, 221–228
April 12, 2016 ©2016 The Authors
http://dx.doi.org/10.1016/j.celrep.2016.03.031

CellPress

OPEN ACCESS
CellPress

Mesolimbic Dopamine Encodes Prediction Errors in a State-Dependent Manner

Georgios K. Papageorgiou,[1,*] Mathieu Baudonnat,[1] Flavia Cucca,[1,2] and Mark E. Walton[1,*]
[1]Department of Experimental Psychology, University of Oxford, 9 South Parks Road, Oxford OX1 3UD, UK
[2]Department of Biomedical Sciences, University of Cagliari, Via Ospedale 72, 09124 Cagliari, Italy
*Correspondence: georgios.papageorgiou@psy.ox.ac.uk (G.K.P.), mark.walton@psy.ox.ac.uk (M.E.W.)
http://dx.doi.org/10.1016/j.celrep.2016.03.031

SUMMARY

Mesolimbic dopamine encodes the benefits of a course of action. However, the value of an appetitive reward depends strongly on an animal's current state. To investigate the relationship between dopamine, value, and physiological state, we monitored sub-second dopamine release in the nucleus accumbens core while rats made choices between food and sucrose solution following selective satiation on one of these reinforcers. Dopamine signals reflected preference for the reinforcers in the new state, decreasing to the devalued reward and, after satiation on food, increasing for the valued sucrose solution. These changes were rapid and selective, with dopamine release returning to pre-satiation patterns when the animals were re-tested in a standard food-restricted state. Such rapid and selective adaptation of dopamine-associated value signals could provide an important signal to promote efficient foraging for a varied diet.

INTRODUCTION

The phasic activity of midbrain dopamine neurons and dopamine release in regions such as the nucleus accumbens (NAc) signal predictions of future reward and discrepancies between such predictions and received reward (Gan et al., 2010; Montague et al., 1996; Schultz et al., 1997; Syed et al., 2016). These signals appear encoded on a common value scale, integrated across different reward attributes, that reflects individuals' subjective preference for particular outcomes rather than the objective properties of reward (Lak et al., 2014). However, such preferences are not fixed but, instead, depend on an organism's current nutritional needs, particularly in comparison with recent consumption. Several studies have shown that dopamine levels in the presence of reward are influenced by current physiological state, as well as the nutritional content of reinforcers (Ahn and Phillips, 1999; Bassareo and Di Chiara, 1999; Beeler et al., 2012; de Araujo et al., 2013; McCutcheon et al., 2012). Nonetheless, to date, the relationship between phasic dopamine, reward prediction errors, nutritional needs, and reward-guided choice remains poorly understood. Here, we investigated this issue by recording dopamine release while rats made choices between food and sucrose solution either in a baseline food-restricted state or after selective satiation on one of the two reinforcers (Rolls et al., 1983). Thus, by monitoring how patterns of dopamine release updated between the sessions, we could investigate how dopamine prediction errors are influenced by selective changes in subjective value and how value predictions and behavioral preferences updated with experience of the reinforcers in a new state.

RESULTS

Behavioral Performance before and after Selective Satiation

Food-restricted rats were trained to perform a two-option operant decision-making task where the selection of each option was associated with a particular type of reward (food pellet or sucrose solution) (Figure 1A). Sessions consisted of trials where only one reward type was available ("forced" trials) and others where rats could choose between the two ("choice" trials). After acquiring the task, the rats (n = 8) performed four sessions: two baseline sessions (A and B), each of which preceded a devaluation session (the devalue food session and the devalue sucrose solution session, order counterbalanced across animals) that was identical to the baseline sessions, except that the rats had free access to one of the rewards for an hour before the test session.

In the first pre-devaluation baseline session (baseline A), the group of rats overall displayed no overall preference in general for either reward type on choice trials (t test against 50% for food choices: $t(7) = 0.168$, $p = 0.87$), no difference in response latencies to the two options on forced trials ($t(6) = 0.94$, $p = 0.38$; the data from one animal was lost because of a computer error), and no differences in the numbers of wrong-lever choices or missed trials (both <2% of trials, $t(7) < 1.60$, $p > 0.15$).

Prior to the devaluation sessions, the rats consumed, on average, either 11.5 g (SEM, ±1.24 g) of pellets or 24.75 ml (SEM, ±1.81 ml) of sucrose solution. This manipulation reliably altered the animals' preference for the reward types (t test against 50% for food choices: devalue food, $t(7) = -2.84$, $p = 0.01$; devalue sucrose solution, $t(7) = 4.13$, $p < 0.01$) (Figure 1B). There was no difference in the magnitude of this change following satiation with either the food or the sucrose solution ($t(7) = 0.29$, $p = 0.78$). There was also a significant increase in the number of missed trials and wrong-lever choices on forced

This is an open access article under the CC BY license (http://creativecommons.org/licenses/by/4.0/).

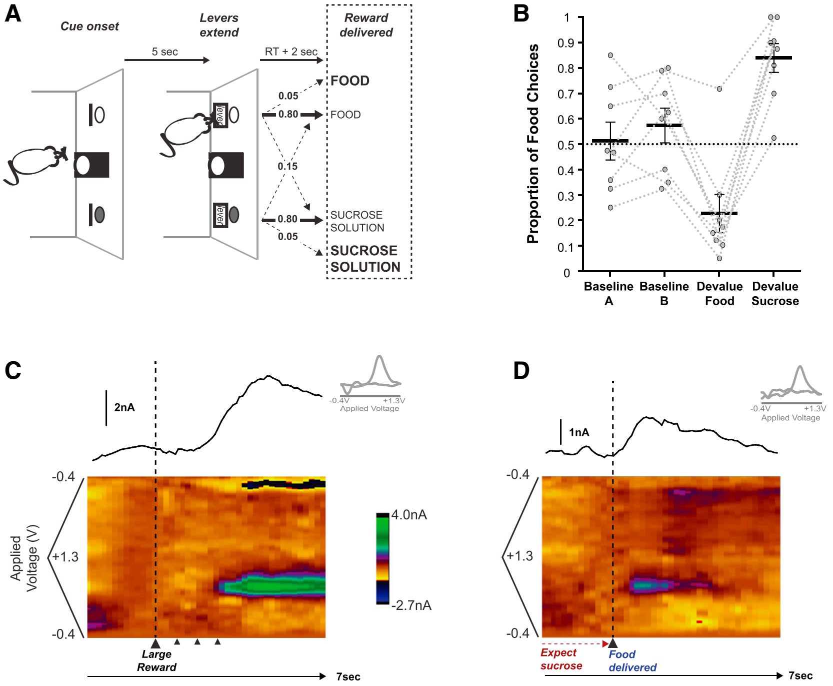

Figure 1. Task Design, Behavioral Performance, and Example Dopamine Signals

(A) Schematic of a typical forced trial (“Forced Left”). Arrows between “Levers extend” and “Reward delivered” indicate the transition probabilities following a response on that option (“FOOD” and “SUCROSE SOLUTION” in regular type indicates standard reward; in bold type, they indicate increased reward). RT, response time.

(B) Proportion of food choices on choice trials (circles correspond to individual rats).

(C and D) Individual example MORE (C) and SWITCH (D) trials. Each panel depicts the recorded current × applied voltage in a pseudocolor plot from 2 s before and 5 s after reward delivery. The upper trace depicts the extract dopamine signal, along with an example cyclic voltammogram identifying the detected current as dopamine.

All averages indicate mean ± SEM.

trials in the devaluation sessions compared to baseline sessions (main effect of devaluation, both $Fs(1, 7) > 7.02$, $p < 0.034$), an effect driven by a selective increase on the devalued option (interaction between reward type and devaluation session: wrong choices, $F(1, 7) = 9.75$, $p = 0.02$; missed trials, $F(1, 7) = 5.53$, $p = 0.051$).

These changes in subjective valuation were temporary and specific to the devaluation session. Preference returned to indifference in the baseline B session run in between the counterbalanced devaluation sessions ($t(7) = 1.08$, $p = 0.32$), and there was no change in choices from the pre-devaluation baseline session ($t(7) = 1.18$, $p = 0.28$).

Dopamine Release at Reward Delivery following Sensory-Specific Satiation Procedures

We monitored dopamine release in the NAc core (Figure S1) using fast-scan cyclic voltammetry while rats performed this reward identity decision paradigm. On 80% of trials, the choice of one lever resulted in the delivery of the standard amount of the expected reward type (“standard” trials). However, on the remaining subset of trials, the animals received either (1) an increased quantity of the expected reward type (value surprise “MORE” trials) or (2) the standard amount of the other reward type (identity surprise “SWITCH” trials) (Figures 1A, 1C, and 1D). Note that, until the reward is dispensed, surprise trials are otherwise identical to standard trials. While we recorded dopamine release in both baseline and selective satiety sessions, here we will mainly focus on patterns of dopamine release in the latter.

To examine the effect of selective satiety and, consequently, a selective change in the subjective value of one of the options, on value-related dopamine signals at the time of reward delivery, we ran a linear regression on the two devaluation sessions (Figures 2A and 2B). There was a strong influence of MORE trials on dopamine release, as well as a significant interaction between MORE trials and reward type. Importantly, the sign of the interaction term switched depending on whether food or sucrose solution was devalued (Figure 2C). This demonstrates reinforcer-specific satiety effects on value surprise trials. The same influence of selective satiety was also observed on SWITCH trials. In sucrose solution devaluation sessions, there was a transient increase in dopamine following the surprise delivery of a valued food pellet after a response on the sucrose solution lever (Figures 2A, 2D, 2F, and 2G). These signals were significantly more discriminable than during the baseline session (paired t test on the dopamine discrimination index: $t(7) = 2.88$, $p = 0.028$). The opposite pattern was observed in the food devaluation sessions: now, it was the surprising delivery of the valued sucrose solution that caused a selective increase in dopamine release, whereas there was no observable increase following a surprise pellet delivery (Figures 2B and 2E–2G). Therefore, surprise-evoked dopamine release can also be modulated by the current state-based value of the reinforcers, demonstrating that the pattern of dopamine is distinct from the physical properties of the reward.

It was also evident that dopamine release differed even on the standard trials for the valued and devalued options in a new state, even though the anticipated type of reward was always delivered ($p < 0.05$; Figures 2D and 2E). To investigate what might be influencing this, we directly contrasted dopamine time locked to reward delivery in the devaluation sessions against an equivalent period recorded in the baseline session (Figure 2H). Surprisingly, there was no consistent difference in the change in average dopamine levels after receipt of the *devalued* reward when compared to receiving that same reward in the baseline session ($p > 0.05$). Instead, there was a small but significant increase in dopamine when receiving the *valued* option for both reinforcers when compared to the same situation during baseline testing (Figure 2H; Figure S2). Moreover, when

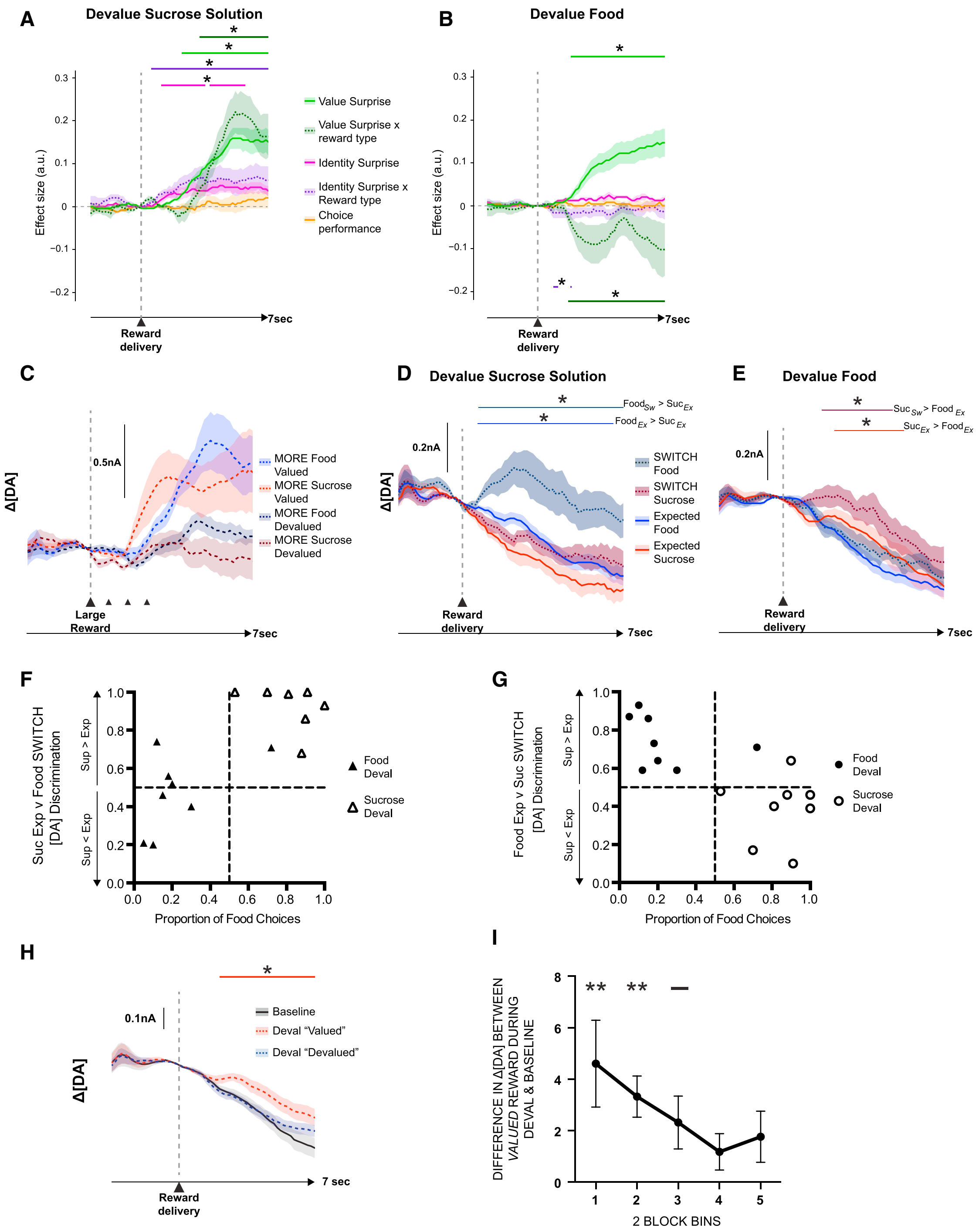

Figure 2. Dopamine at Reward Delivery after Selective Satiation

(A and B) Average effect sizes from a general linear model of post-reward dopamine signals after sucrose solution (A) or food (B) devaluation.

(C) Average dopamine release on MORE food or sucrose trials divided up by the reward type that was devalued prior to the session.

(D and E) Dopamine signals on expected (Exp) and SWITCH (Sw) trials after sucrose (Suc) (D) or food (E) was selectively devalued.

(F and G) Dopamine discrimination index for each animal in a 5 s post reward window for SWITCH food versus expected sucrose (F) or SWITCH sucrose versus expected food (G) plotted against each animal's food choices. Data are separated into food (filled symbols) or sucrose solution (open symbols) devaluation sessions.

(H) Comparison of dopamine signals when receiving expected reward in baseline and devaluation sessions as a function of which reward type was devalued.

(legend continued on next page)

the session was divided into five blocks, this difference was found to be, on average, largest at the beginning of the session and diminished linearly as the session progressed (linear main effect of block: $F(1, 7) = 5.88$, $p = 0.046$) (Figure 2I). Therefore, receipt of the valued reward following selective satiety procedures appeared to produce a small positive prediction error that updated as the animals gained more experience of the reward in the new state.

Rapid Updating of Cue-Elicited Dopamine Release after Changes in State

The pattern of dopamine release at reward delivery suggests that value predictions are shaped by current motivational state in a reinforcer-specific manner. If so, state-based modulations of value predictions should also be observable at cue onset.

As can be observed in Figure 3, this is exactly what we found. While food cues elicited significantly greater dopamine release than sucrose solution cues in the sucrose solution devaluation session (Figure 3A), this reversed after food devaluation, with dopamine release for food cues now lower than after sucrose solution cues (Figure 3B). This was borne out by a linear regression that showed a significant effect of reward type on cue-evoked dopamine in both sessions, but with the sign modulated by the identity of the pre-fed reinforcer (Figures 3C and 3D). To further investigate this change in cue-elicited release, dopamine levels either on forced food or forced sucrose solution trials were analyzed across five equally sized blocks in the session. This showed that these effects occurred rapidly, being evident within the first block of the session (analysis of valued or devalued cue dopamine: $F(1, 6) = 11.93$, $p = 0.014$; no main effect or interaction with reward type: both $Fs < 1.39$, $p > 0.28$; $n = 7$, as one animal was excluded for having $\leq 5\%$ responses on the devalued option) (Figure 3E).

Interestingly, although selective satiation uniformly decreased dopamine after *devalued* cues across the session, there was an asymmetric effect on *valued* cue-elicited dopamine (Figures 3F and 3G). Specifically, after food devaluation, dopamine levels were, on average, significantly greater after sucrose solution cues compared to baseline sessions ($p < 0.05$). By contrast, there was no statistically reliable change in either direction in response to valued food cues after sucrose solution devaluation. In other words, after eating to satiety, the predicted value of the sweet liquid option increased. Nonetheless, while the selective satiety procedures reliably biased choice behavior and modulated dopamine release, there was no observable consistent relationship between the size of cue-elicited signals in a particular session for a particular animal and its preference for one reinforcer over the other on choice trials (Figures 3C–3E and 3H).

Although these analyses show a rapid influence of selective satiety on dopamine release, it is not clear whether this is purely an experience-dependent effect based on learning the value of the options in the new state or whether dopamine cue signals can update even before the devalued reward is consumed during the session. To examine this, we analyzed dopamine release elicited by the first presentation in the session of both the valued and devalued options (Figure 4A). This revealed an overall attenuation of dopamine release on the initial trial of the devaluation sessions compared to the preceding baseline session (main effect of session type: $F(1, 6) = 12.44$, $p = 0.012$, including trial order as a between-subjects factors). However, this reduction was not significantly greater after first presentation of the cue associated with the currently devalued option than after that associated with the currently valued option (interactions including Session Type × Devaluation: all $Fs < 0.27$, $p > 0.62$). This implies that selective satiety induces an immediate general, rather than stimulus-specific, reduction in cue-elicited dopamine signals but that rats need experience of the reinforcer in the new state to fully update learned cue associations.

Nonetheless, inspection of Figure 4A suggests that dopamine levels were, on average, lower on the first devalued trial compared to cue presentations in valued states, particularly in the later period between lever extension and reward delivery. Therefore, we also directly compared average dopamine levels during the lever extension/response period, prior to reward delivery, on the first valued and devalued trials. This confirmed that dopamine levels were significantly attenuated after presentation of the devalued lever, compared to after the valued lever, even though the reinforcers had yet to be directly experienced in the new state (main effect of devaluation: $F(1, 6) = 12.63$, $p = 0.012$) (Figure 4B). This occurred in spite of the fact that there were no differences in lever press latency between the first valued or devalued trial (mean ± SEM: valued, 0.42 s ± 0.09 s; devalued, 0.60 s ± 0.20 s; $t(7) = 1.05$, $p = 0.33$).

Importantly, although selective satiation strongly modulated dopamine levels in the devaluation session, this did not have a lasting influence over patterns of dopamine release. The first presentation of the previously devalued option in baseline B immediately elicited comparable levels of dopamine as when that same cue had been presented during the first baseline A session (comparison between first trial dopamine in baseline A and baseline B, separated by which option was devalued during devaluation A: all $Fs < 0.96$, $p > 0.36$) (Figure 4C). Therefore, while dopamine signals update with experience of the reinforcer in the new state, they immediately revert to the original learned values once animals return to a baseline food-restricted state.

DISCUSSION

These results demonstrate that mesolimbic dopamine flexibly encodes reward prediction error signals shaped by the specific properties of a reward to satisfy a current need. Midbrain dopamine neurons in primates tested for multiple days in a similar state have been shown to encode reward prediction errors that reflect the animals' subjective preference for different reward

(I) Difference between average dopamine release after reward delivery when receiving the valued option in devaluation sessions and this same reward type in the previous baseline session (collapsed over reinforcers), divided into five bins each of two blocks of trials.
Lines: *$p < 0.05$ permutation tests, corrected for multiple comparisons; **$p < 0.05$; $^{-}$ $p = 0.058$, two-tailed t test against 0. All averages indicate mean ± SEM. DA, dopamine.

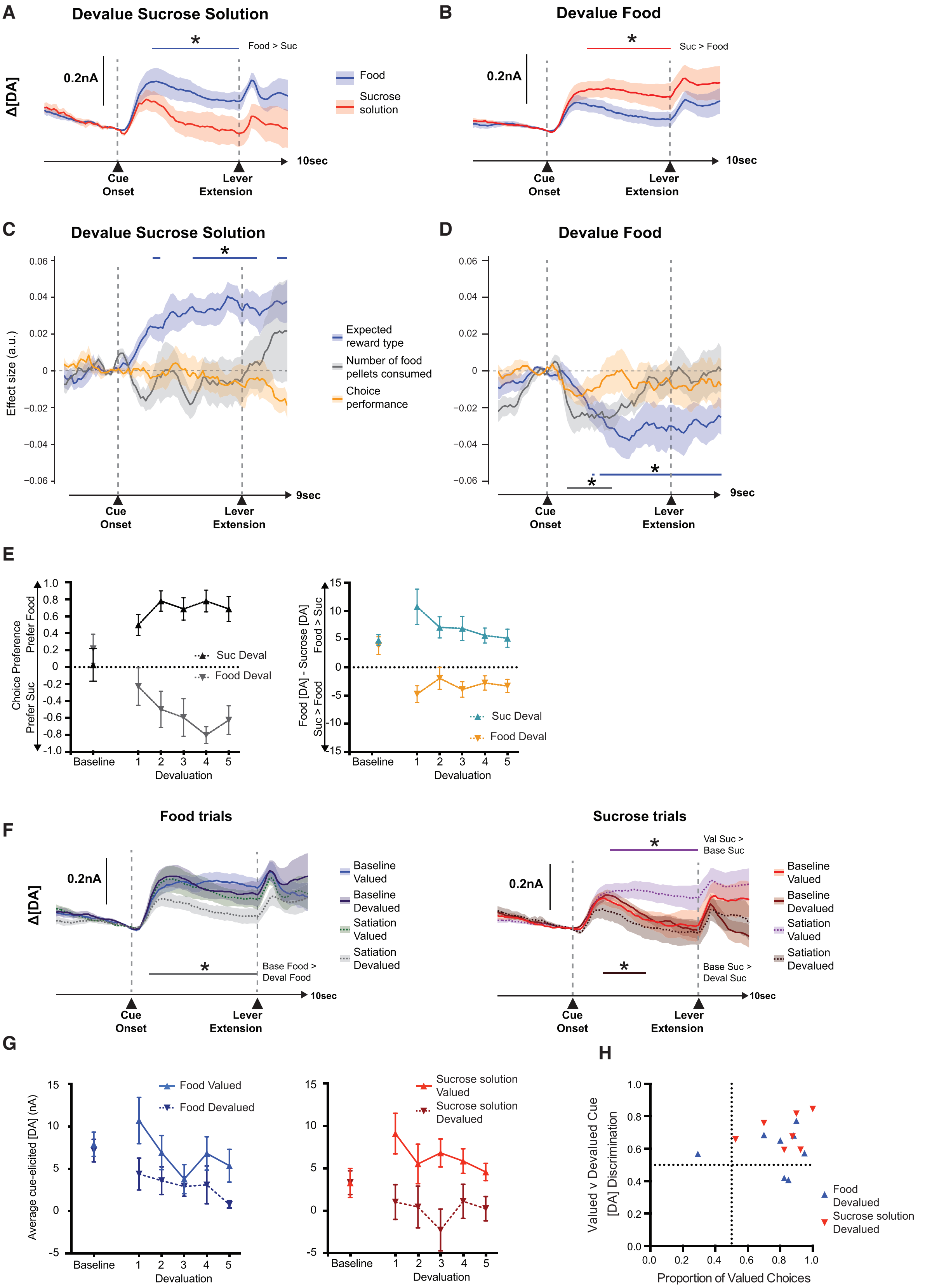

(legend on next page)

OPEN
ACCESS
CellPress

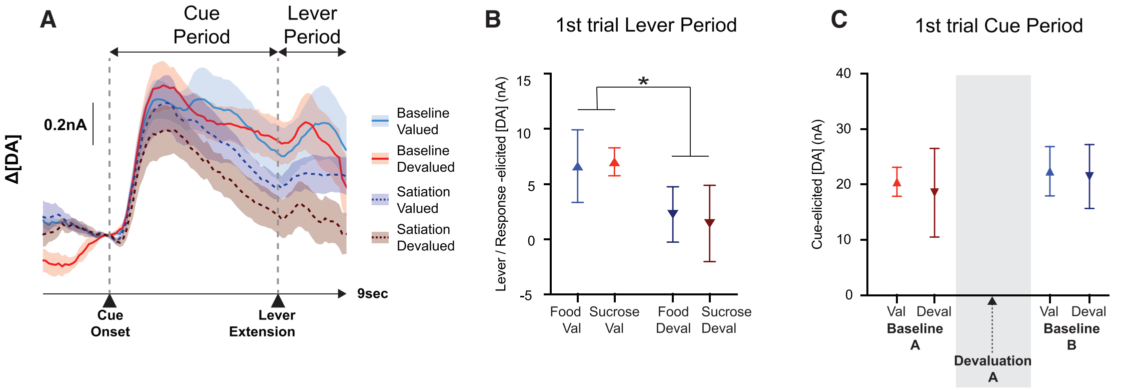

Figure 4. Cue-Elicited Dopamine Release on First Trials of the Session

(A) Average dopamine levels in a 5-s window after cue onset on the first food/sucrose solution trial in baselines A and B. Baseline data are divided into "valued" (Val) or "devalued" (Deval) based on which reinforcer the animals had free access to in devaluation A.

(B) Average dopamine signals after cue onset on the first food/sucrose solution trial in the baseline and devaluation sessions. Baseline data here are divided up based on which reinforcer the animals had free access to in the subsequent devaluation session.

(C) Average dopamine levels in a 2-s post-lever extension window (prior to reward delivery) on the first food or sucrose solution trial, averaged across the devaluation sessions.

Levels were significantly reduced on the first devalued trial compared to the first valued trial (*$p < 0.05$, ANOVA). All averages indicate mean ± SEM. DA, dopamine.

types (Lak et al., 2014). Here, we observed a rapid, experience-driven updating of NAc core dopamine signals, both to predictive cues and reward delivery, to reflect the subjective value of stimuli following selective satiation.

Several of our results, therefore, appear consistent with key predictions of model-free temporal difference learning models. Dopamine release on SWITCH trials in the devaluation sessions principally encoded surprising changes in reward identity based on discrepancies between expected and received value rather than the sensory surprise of receiving the alternative reinforcer. This does not rule out that coding of reward identity prediction errors may exist in other contexts, where the value difference between the options is less prominent or when a change in identity is more relevant for behavior. For instance, the SWITCH trials here occurred as rare fluctuations in an otherwise stable task, but in other paradigms, such as unblocking or reversal learning, a change in reward identity can be more long lasting and of more significance for behavior (McDannald et al., 2014; Stalnaker et al., 2014). Equally, it is possible that distinct dopamine pathways might contain additional information about reward identity or other aspects of reward (Huetteroth et al., 2015). The current data were collected from the NAc core, as dopamine release in this structure has been shown to signal discrepancies from expectation (Day et al., 2007; Syed et al., 2016). However, in rodents, the NAc shell rather than the core—and, specifically, the D1-receptor-expressing medium spiny neurons in this region—has been associated with the ability of specific reinforcers to motivate and invigorate responding and promote feeding (Corbit and Balleine, 2011; Laurent et al., 2014; O'Connor et al., 2015).

We observed a strong modulation of both food- and sucrose-solution-elicited dopamine by the amount of reinforcer consumed within and prior to the session. Such selective modulation of stored value signals by specific satiety may be important to promote efficient and varied foraging behaviors. Cue-elicited dopamine release rapidly updated, with significant differences between the valued and devalued signals being evident within

Figure 3. Dynamic Changes in Cue-Evoked Dopamine after Selective Satiation

(A–D) Average cue-evoked dopamine (DA) signals (A and B) or effect sizes from a general linear model (C and D) after selective satiation on sucrose solution (Suc) (A and C) or food (B and D).

(E) Change in preference and relative cue-evoked dopamine plotted over five bins each of two blocks of trials following sucrose solution or food devaluation. Choice is expressed as a change from 50% [(2 × proportion of food choices) − 1]. Relative cue-evoked dopamine is the difference between average food cue and sucrose solution cue dopamine levels during the 5-s post-cue period. The average difference across the whole of the immediately preceding baseline session is presented for comparison.

(F) Comparison between average cue-evoked dopamine release during the baseline and devaluation sessions on food (left) or sucrose solution trials (right). Data are divided up into "valued" and "devalued" based on which reinforcer the rats had free access to before the devaluation session. Baseline data are from the immediately preceding baseline session.

(G) Average dopamine in the 5-s post-cue period in the devaluation sessions, divided up into five bins each of two blocks of trials. The average difference across the whole of the immediately preceding baseline session is presented for comparison.

(H) Dopamine discrimination index for each animal in the 5 s post cue period for both valued food versus devalued sucrose solution (red triangles) and for valued sucrose solution versus devalued food (blue triangles), plotted against each animal's choices of the valued reinforcer in that session. There was no reliable relationship between these measures ($r = 0.183$, $p = 0.51$).

Lines: *$p < 0.05$ permutation tests, corrected for multiple comparisons. All averages indicate mean ± SEM.

the first block of trials following selective satiation. However, this appeared to be predominantly shaped by direct incentive learning in the new state. While there was a general reduction in dopamine on the first trials of the devaluation sessions, compared to the preceding baseline sessions, this was not selective for the devalued option. This is in line with studies showing a general activating role for the NAc core, and dopamine transmission in this region, in the presence of reward-associated cues to motivate and invigorate available actions (Corbit and Balleine, 2011; du Hoffmann and Nicola, 2014).

However, it is notable that there were already selective differences in dopamine levels in the period after lever extension while the rat was making a response and waiting for either the valued or devalued reward. Therefore, some aspects of dopamine signaling can partially update without direct experience of the outcome (Bromberg-Martin et al., 2010). Moreover, cue-elicited dopamine returned to pre-devaluation patterns by the start of the subsequent baseline session run in a food-restricted state, in spite of the large difference between release elicited by the valued and devalued cues at the end of the devaluation session. This implies that mesolimbic dopamine systems have access to stored memories of learned incentive values when returning to a familiar state.

Together, our data add to the evidence indicating a close link between mesolimbic dopamine and physiological state (de Araujo et al., 2012; McCutcheon, 2015; Sclafani et al., 2011). Ventral tegmental area dopamine neurons receive excitatory inputs from the lateral hypothalamus (Watabe-Uchida et al., 2012) and dopamine cell activity, and NAc core dopamine release is influenced by physiological state (Branch et al., 2013) and by peptides involved in appetite (Cone et al., 2014). In the present experiment, decisions will be made based not only on the objective sensory qualities of a food pellet versus a bolus of sucrose solution but also on their subjective value in a given state. The rapid adaptation of mesolimbic dopamine signals following a change in state would potentially allow it to play an important role in prioritizing behaviors based on the available opportunities.

EXPERIMENTAL PROCEDURES

Animals

17 male Sprague-Dawley rats were used for this experiment, of which 8 contributed data reported here (see the Supplemental Experimental Procedures). During the training and testing periods, access to food was restricted so that rats' weights were kept between 85% and 90% of their free-feeding body weight. Water was continuously available in the home cages. All procedures were in compliance with the United Kingdom Animals Scientific Procedures Act of 1986 and the University of Oxford Policy on the Use of Animals in Scientific Research. All experiments were approved by the University of Oxford Animal Welfare and Ethical Review Board.

Behavioral Paradigm

We used fast-scan cyclic voltammetry to record dopamine release from chronically implanted carbon fiber electrodes in the NAc, as described previously (Clark et al., 2010; Syed et al., 2016), as animals performed a two-option/two-reward decision-making task. Sessions consisted of 120 trials, broken down into blocks of eight forced trials (four to each lever in a pseudorandom order) followed by four free-choice trials. One option was consistently associated with one reward type (45-mg food pellet), and the other was associated with a bolus of sucrose liquid (95 μl 20% sucrose solution), both delivered to the same food cup. On 80% of trials, animals received the reinforcer associated with the selected option. However, on 10% of the forced trials and 5% of the choice trials, the animals unexpectedly received the reward associated with the other lever ("SWITCH"). On another 5% of the forced trials, the animals received four times more reward than expected, although of the expected identity ("MORE"). These surprise trials occurred pseudorandomly throughout the session.

Data Analysis

As in previous studies, dopamine signals were extracted using principal-component analysis (Heien et al., 2004; Syed et al., 2016). To quantify which factors affected dopamine levels, regression coefficients were estimated for each animal at each time point around an event of interest. A linear model was used with a constant term, representing an ordinary least-squares fit of the given regressors to the data over trials (see the Supplemental Experimental Procedures). The discriminability of dopamine signals in pairs of different trial types was analyzed in each individual animal at each time point using the area under the receiver operating characteristic curve (auROC) (Syed et al., 2016). All data are reported as significant based on permutation tests when $p < 0.05$, corrected for multiple comparisons (i.e., $p < 0.001$). To calculate a dopamine discriminability index, we calculated the auROC using the average dopamine in the 5-s window after reward delivery for a particular trial type. In situations where there were insufficient numbers of trials to calculate an auROC (i.e., when examining changes in bins of trials across the session), we extracted the average dopamine levels instead, within a 3-s window after reward delivery, a 5-s window between cue onset and lever extension, or a 2-s window between lever extension and reward delivery, and performed a repeated-measures ANOVA.

SUPPLEMENTAL INFORMATION

Supplemental Information includes Supplemental Experimental Procedures and two figures and can be found with this article online at http://dx.doi.org/10.1016/j.celrep.2016.03.031.

AUTHOR CONTRIBUTIONS

G.K.P. and M.E.W. conceived the study; G.K.P. and M.B. performed surgeries; G.K.P. collected the data with the assistance of F.C.; M.E.W. and G.K.P. analyzed the data; and M.E.W. and G.K.P. wrote the manuscript.

ACKNOWLEDGMENTS

This work was funded by a Wellcome Trust Research Career Development Fellowship (WT090051MA to M.E.W.). G.K.P. received support from the A.G. Leventis Foundation. We would like to thank Emilie Syed for advice about data analysis, Greg Daubney for histology, and Matthew Rushworth for initial discussions about paradigm design.

Received: June 2, 2015
Revised: February 14, 2016
Accepted: March 8, 2016
Published: March 31, 2016

REFERENCES

Ahn, S., and Phillips, A.G. (1999). Dopaminergic correlates of sensory-specific satiety in the medial prefrontal cortex and nucleus accumbens of the rat. J. Neurosci. *19*, RC29.

Bassareo, V., and Di Chiara, G. (1999). Modulation of feeding-induced activation of mesolimbic dopamine transmission by appetitive stimuli and its relation to motivational state. Eur. J. Neurosci. *11*, 4389–4397.

Beeler, J.A., McCutcheon, J.E., Cao, Z.F., Murakami, M., Alexander, E., Roitman, M.F., and Zhuang, X. (2012). Taste uncoupled from nutrition fails to sustain the reinforcing properties of food. Eur. J. Neurosci. *36*, 2533–2546.

Branch, S.Y., Goertz, R.B., Sharpe, A.L., Pierce, J., Roy, S., Ko, D., Paladini, C.A., and Beckstead, M.J. (2013). Food restriction increases glutamate receptor-mediated burst firing of dopamine neurons. J. Neurosci. *33*, 13861–13872.

Bromberg-Martin, E.S., Matsumoto, M., Hong, S., and Hikosaka, O. (2010). A pallidus-habenula-dopamine pathway signals inferred stimulus values. J. Neurophysiol. *104*, 1068–1076.

Clark, J.J., Sandberg, S.G., Wanat, M.J., Gan, J.O., Horne, E.A., Hart, A.S., Akers, C.A., Parker, J.G., Willuhn, I., Martinez, V., et al. (2010). Chronic microsensors for longitudinal, subsecond dopamine detection in behaving animals. Nat. Methods *7*, 126–129.

Cone, J.J., McCutcheon, J.E., and Roitman, M.F. (2014). Ghrelin acts as an interface between physiological state and phasic dopamine signaling. J. Neurosci. *34*, 4905–4913.

Corbit, L.H., and Balleine, B.W. (2011). The general and outcome-specific forms of Pavlovian-instrumental transfer are differentially mediated by the nucleus accumbens core and shell. J. Neurosci. *31*, 11786–11794.

Day, J.J., Roitman, M.F., Wightman, R.M., and Carelli, R.M. (2007). Associative learning mediates dynamic shifts in dopamine signaling in the nucleus accumbens. Nat. Neurosci. *10*, 1020–1028.

de Araujo, I.E., Ferreira, J.G., Tellez, L.A., Ren, X., and Yeckel, C.W. (2012). The gut-brain dopamine axis: a regulatory system for caloric intake. Physiol. Behav. *106*, 394–399.

de Araujo, I.E., Lin, T., Veldhuizen, M.G., and Small, D.M. (2013). Metabolic regulation of brain response to food cues. Curr. Biol. *23*, 878–883.

du Hoffmann, J., and Nicola, S.M. (2014). Dopamine invigorates reward seeking by promoting cue-evoked excitation in the nucleus accumbens. J. Neurosci. *34*, 14349–14364.

Gan, J.O., Walton, M.E., and Phillips, P.E. (2010). Dissociable cost and benefit encoding of future rewards by mesolimbic dopamine. Nat. Neurosci. *13*, 25–27.

Heien, M.L., Johnson, M.A., and Wightman, R.M. (2004). Resolving neurotransmitters detected by fast-scan cyclic voltammetry. Anal. Chem. *76*, 5697–5704.

Huetteroth, W., Perisse, E., Lin, S., Klappenbach, M., Burke, C., and Waddell, S. (2015). Sweet taste and nutrient value subdivide rewarding dopaminergic neurons in Drosophila. Curr. Biol. *25*, 751–758.

Lak, A., Stauffer, W.R., and Schultz, W. (2014). Dopamine prediction error responses integrate subjective value from different reward dimensions. Proc. Natl. Acad. Sci. USA *111*, 2343–2348.

Laurent, V., Bertran-Gonzalez, J., Chieng, B.C., and Balleine, B.W. (2014). δ-opioid and dopaminergic processes in accumbens shell modulate the cholinergic control of predictive learning and choice. J. Neurosci. *34*, 1358–1369.

McCutcheon, J.E. (2015). The role of dopamine in the pursuit of nutritional value. Physiol. Behav. *152* (*Pt B*), 408–415.

McCutcheon, J.E., Beeler, J.A., and Roitman, M.F. (2012). Sucrose-predictive cues evoke greater phasic dopamine release than saccharin-predictive cues. Synapse *66*, 346–351.

McDannald, M.A., Esber, G.R., Wegener, M.A., Wied, H.M., Liu, T.L., Stalnaker, T.A., Jones, J.L., Trageser, J., and Schoenbaum, G. (2014). Orbitofrontal neurons acquire responses to 'valueless' Pavlovian cues during unblocking. eLife *3*, e02653.

Montague, P.R., Dayan, P., and Sejnowski, T.J. (1996). A framework for mesencephalic dopamine systems based on predictive Hebbian learning. J. Neurosci. *16*, 1936–1947.

O'Connor, E.C., Kremer, Y., Lefort, S., Harada, M., Pascoli, V., Rohner, C., and Lüscher, C. (2015). Accumbal D1R Neurons Projecting to Lateral Hypothalamus Authorize Feeding. Neuron *88*, 553–564.

Rolls, E.T., Rolls, B.J., and Rowe, E.A. (1983). Sensory-specific and motivation-specific satiety for the sight and taste of food and water in man. Physiol. Behav. *30*, 185–192.

Schultz, W., Dayan, P., and Montague, P.R. (1997). A neural substrate of prediction and reward. Science *275*, 1593–1599.

Sclafani, A., Touzani, K., and Bodnar, R.J. (2011). Dopamine and learned food preferences. Physiol. Behav. *104*, 64–68.

Stalnaker, T.A., Cooch, N.K., McDannald, M.A., Liu, T.L., Wied, H., and Schoenbaum, G. (2014). Orbitofrontal neurons infer the value and identity of predicted outcomes. Nat. Commun. *5*, 3926.

Syed, E.C., Grima, L.L., Magill, P.J., Bogacz, R., Brown, P., and Walton, M.E. (2016). Action initiation shapes mesolimbic dopamine encoding of future rewards. Nat. Neurosci. *19*, 34–36.

Watabe-Uchida, M., Zhu, L., Ogawa, S.K., Vamanrao, A., and Uchida, N. (2012). Whole-brain mapping of direct inputs to midbrain dopamine neurons. Neuron *74*, 858–873.

Cell Reports, Volume *15*

Supplemental Information

Mesolimbic Dopamine Encodes Prediction Errors in a State-Dependent Manner

Georgios K. Papageorgiou, Mathieu Baudonnat, Flavia Cucca, and Mark E. Walton

Supplementary Experimental Procedures

Animals

17 male Sprague-Dawley rats weighing 350-400g were used for this experiment (Harlan Olac, Bicester, UK). This included 11 naïve rats aged 3-6 months at the start of testing as well as an additional 6 rats, aged 6 months, which had previously participated in a separate 2-option appetitive decision making experiment. A total of 6 rats were excluded from the electrochemistry analysis, 3 for misplaced electrodes outside the nucleus accumbens core and 3 for broken/noisy electrodes. In addition, 3 rats did not perform the Devaluation sessions and so are not included in the analyses here, leaving a total of 8 rats. As we wanted to relate dopamine release to behaviour, we have only included data from 1 electrode in each individual. Animals were maintained on a 12-hr light/dark cycle, were initially grouped housed in cages of 3 but then were individually housed after surgery and during the testing period. All testing was carried out during the light phase. During the training and testing periods, access to food was restricted such that rats' weights were kept between 85-90% of their free-feeding body weight. Water was continuously available in the home cages. All procedures were in compliance with the United Kingdom Animals Scientific Procedures Act (1986).

Surgical Procedures

Animals were anaesthetised using isoflurane (4% induction and 1.5% for maintenance) and given buprenorphine (Vetergesic, 0.1 ml/kg) at the start of the surgical procedure. Body temperature was maintained at 37±0.5 C with the use of a homeothermic heating blanket. Corneal dehydration was prevented with application

of ophthalmic ointment (Lacri-Lube, Allergan, UK). After induction, the rat's head was shaved and secured in a stereotaxic frame. The head was then cleaned with dilute hibiscrub, 70% alcohol and a local anaesthetic, bupivacaine, was applied to the area. The scull was then exposed and holes were drilled for an Ag/AgCl reference electrode (AP: -3.7, ML: -1.4), 4 anchoring screws (Precision Technology Supplied Ltd, UK) and a recording electrode in each hemisphere. After the screws were secured and the reference electrode inserted, custom-made carbon fibre microelectrodes were then lowered into the NAc core (AP: +1.4, ML: ±1.3, DV: 7.0). The carbon fibre microelectrodes and reference electrode were attached to a headstage connector, which was secured in place along with an anchoring headpost using dental cement (Kemdent, Swindon, UK). Following surgery, animals were administered additional buprenorphine (0.1 ml/kg) and meloxicam (Metacam, 0.2 ml/kg). Meloxicam was also administered for at least 3 days following surgery. Animals had on average three weeks of post-surgery recovery with food and water *ad libitum*, prior to food restriction and further behavioural training/recording.

Behavioural Paradigm

Apparatus

Testing was carried out in custom-designed operant chambers (30.5 x 24.1 x 29.2 cm; Med Associates, VT, USA). Each chamber was housed within a sound-attenuating cabinet ventilated with a fan, which provided constant background noise of ~64dB. Each chamber contained two retractable levers, situated 9.5cm on either side of a reward magazine that contained a receptacle into which both 45mg standard grain-based pellets (Test Diet, distributed through Sandown Scientific, UK)

and sucrose solution could be delivered. Above each lever there was a cue light. The magazine was fitted with an infrared beam that signalled when animals entered the receptacle. Each chamber was also fitted with a house-light.

Training

Rats were first given experience of each type of reward in the operant recording chamber by placing a number of pellets or amount of sucrose solution in the reward receptacle during two brief sessions separated by ~1 hour (reward order counterbalanced across animals). On the next day, the rats received a magazine training session with both reinforcers. In this session (duration ~30mins), pellets or sucrose solution were delivered on VI60 schedule. Subsequently, rats were taught to press levers to gain reward. Half of the rats were trained that the left lever would lead to sucrose solution and the right lever to pellets, whereas the other half were trained on the opposite configuration. These associations remained fixed throughout the data acquisition. Once the animals were making ~100 responses on both levers, they moved on to the main paradigm.

The main paradigm, used during both behavioural and subsequent voltammetric recording sessions, consisted of individual trials where animals could press a lever to gain one of the two reward types. Sessions could contain 'forced' trials where only one reward type was available, and 'free choice' trials where both reward types were available. At the start of a forced trial, the house light would turn on and one of the two cue lights would illuminate. After a delay of 5s, both levers would extend. A single lever press on the option under the illuminated cue would cause both levers to retract and reward to be delivered to the receptacle 2s later.

The cue light over the selected option remained illuminated during this delay to reduce any working memory load between choice and reward delivery. There was then an inter-trial interval (ITI) of 25 – 35s. A response on the lever under the non-illuminated cue light was counted as an error and did not lead to any reward delivery. If the rat failed to make a response within 10s, this trial was counted as an omission. After either an error or omission, the levers would retract, cue and house lights turn off, and the ITI would immediately start. Choice trials were identical to forced trials except that both cue lights would illuminate and selection of either option within 10s would result in reward delivery 2s after this choice. The houselight was on throughout the session.

Reward value titration

To establish the association between each response option and a specific reward during titration sessions, the rats were first given a session with only forced trials (40 to each lever in a pseudorandom order). They then had a session where forced trials were interspersed with equal numbers of choice trials, which could be used to determine each animal's preference for the reward types. Each session consisted on 80 trials made up of blocks of 4 forced trials (2 to the left, 2 to the right lever, pseudorandom order) followed by 4 choice trials. Based on pilot data in separate animals, rats were tested with 95µl of 20% sucrose solution across two separate sessions. Our criteria for determining whether this was an appropriate volume and concentration of sucrose for the group of rats were (a) stable performance across two Pre-Test titration sessions (no significant change across the sessions) and (b) average number of food choices not being significantly different from 50%.

Reward identity task

We used fast-scan cyclic voltammetry to record dopamine release as animals performed a modified version of the 2-choice/2-reward decision-making task described above. All sessions consisted of 120 trials, consisting of 10 blocks of 12 trials (8 forced trials, 4 to each lever in a pseudorandom order, followed by 4 free choice trials). On 80% of trials, animals received the reinforcer associated with the selected option ("expected reward"). However, on 10% of the forced trials and 5% of the choice trials, the animals received the reward associated with the other lever ("SWITCH" trials). On another 5% of the forced trials the animals received four times more reward than expected though of the expected identity ("MORE" trials).

There were 4 separate recording sessions, consisting of 2 baseline sessions and 2 selective satiation sessions. Selective satiation sessions had exactly the same structure with the baseline sessions but with the difference that prior to those sessions animals were given 1 hour's free access to either sucrose solution or food pellets in the testing chamber before the task commenced. During food devaluation, the animals did not have access to any fluids. The session order was always Baseline A – Selective Satiation A – Baseline B – Selective Satiation B, with the order of satiation (food or sucrose solution) counterbalanced across animals.

Fast-scan cyclic voltammetry

Fast-scan cyclic voltammetry (FSCV) recordings were made from chronically-implanted carbon fibre electrodes. Voltammetric scans were performed at a frequency of 10Hz throughout the session. Prior to a scan, the carbon fibre was held

at a potential of -0.4V (vs AG/AgCl) and then, during the scan, ramped up to +1.3V and back to -0.4V at 400 V/sec. The application of this waveform causes redox reactions in electrochemically active species, such as dopamine, at the surface of the carbon fibre that can be recorded as changes in current over time. Based on previously established criteria the recorded current in response to uncued pellet and sucrose delivery, obtained at the start and end of each recording session, was used to determine the chemical sensitivity of the recording electrode to dopamine on that particular session. An extracted cyclic voltammogram was linearly regressed against a dopamine standard, with $r^2 \geq .75$ set as the criterion based on the discriminability of dopamine from other common neurochemicals in a flow cell (Gan et al., 2010).

Data analysis

Each animal's preference for food over sucrose solution was calculated as the number of food choices / (number of food choices + number of sucrose choices). Subjective preference in each session was tested as a 2-tailed t-test against indifference (50%) and consistency across sessions as a repeated measures ANOVA with session as a within-subjects factor. Response latencies were calculated as the time from lever extension to a response.

Voltammetric analysis was initially carried out using software written in LabVIEW (National Instruments). Data were low-pass filtered at 2kHz. To isolate changes in dopamine concentration from other electrochemical signals, a principal component analysis was performed using a standard training set of stimulated dopamine release detected by chronically implanted electrodes, with dopamine treated as the first principal component among other unrelated electrochemical

fluctuations such as changes in pH (Heien et al., 2004). The data were smoothed using a 0.5s moving window. Trials where the PCA failed to successfully extract dopamine current on >50% of data points in a trial were excluded. Once dopamine-related current changes were extracted all further analysis was undertaken using Matlab® (Mathworks, MA, USA).

To quantify which factors affected dopamine levels, regression coefficients were estimated for each animal at each time point in either a 7s window spanning from 2s before reward delivery to 5s after reward or a 9s window from 2s before cue onset to 7s after (cue and lever extension periods). A linear model was used with a constant term, representing an ordinary least-squares fit of the given regressors to the data over trials.

For analysis of the reward-evoked dopamine on all correct trials, the regressors of interest were: (1) SWITCH trial, (2) MORE trial, (3) cumulative number of food pellets consumed, and (4) preference for food in the choice trials at the end of each block (transformed to be 1 for 100% preference for food and –1 for 100% preference for sucrose solution), as well as interaction terms for (5) SWITCH x reward type, (6) MORE x reward type, and (7) preference x reward type. We also included two regressors for reward type (food trials were assigned 1, sucrose solution were assigned –1) and trial type (forced 1, choice –1), though these are not presented to the sake of clarity. For analysis of cue-evoked dopamine on all correct trials, the regressors were again: (1) reward type, (2) trial type, (3) amount of food consumed, and (4) block-by-block food preference, and 3 terms for the interaction between reward type and the other regressors.

Each trial in each regressor was modeled with a single value. All regressors except for choice performance, whether continuous or categorical, were mean-centered. Regression coefficients in each animal were averaged. We focused on the significance of the regression coefficients in the 5s post-reward period or 5s post-cue onset period was tested against a population of 1000 coefficients obtained by randomly permuting the pairings between the regressors and the data (we also ran the post-cue GLM to include the 2s period after lever extension, though these data are not discussed here and statistical analyses were not adjusted to include this period). Permutation tests were considered significant at any time point when the regression coefficient from the real data exceeded the maximum or minimum of the permuted population of coefficients ($p < 0.05$, corrected for multiple comparisons over the 5s after event onset; i.e., $p < 0.001$ uncorrected).

The discriminability of dopamine signals between pairs of trial types (e.g., expected sucrose versus surprise food) was analysed in each individual animal at each time point in a 5s period after either reward delivery or cue onset (between cue onset and lever extension) using the area under the receiver operating characteristic curve (auROC). The auROC from each animal was then averaged and significant discriminability at each time point was determined using 1000 random permutations of the trial types and re-computing the auROC to generate a null distribution. Permutation tests were considered significant at any time point when $p < 0.05$, corrected for multiple comparisons (i.e., $p < 0.001$ across 50 timepoints).

For the Devaluation sessions, we combined the data across the counterbalanced food / sucrose devaluation sessions to look at the effects in the whole group for each manipulation. We here focused on (a) surprise signals

(SWITCH / MORE), but also (b) contrasted dopamine signals on the expected reward trials in sessions depending on whether the reward type was devalued or not (non-devalued is termed “valued” throughout) (e.g., expected valued sucrose versus expected devalued food / expected valued food versus expected devalued sucrose). As there were only a few value surprise trials in each session, it was not possible to directly compare these trial types using the auROC approach. Therefore, instead, we found the average signal in a 3s window, from 0.5-3.5s after reward delivery, and compared these using a repeated measures ANOVA with within subjects factors devaluation session and reward type and devaluation order as a between subjects factor (NB. the results were unchanged if a peak measure was taken instead). To examine changes in dopamine release after a state change on standard trials, we combined the data across reward types in the Selective Satiation sessions into valued and devalued trials and then, using an auROC, compared dopamine on expected reward dopamine on these two trial types against signals recorded in the baseline session. We also did comparable analyses on the data from each reinforcer in isolation. To look at how the valued signals changed during the session, we first broke these sessions down into 5 bins, each containing 2 blocks of trials (i.e., 8 forced food and 8 forced sucrose solution) and subtracted the average dopamine signal in a 0.5-3.5s window after reward delivery on expected reward trials in the Baseline session and the valued expected reward trials during the Devaluation session. To account for variance in the data, we log transformed the values before running a repeated measures ANOVA, with trial bin as a within-subjects factor, and *post-hoc* 1-sample t-tests against zero to determine in which bins there was significantly greater dopamine during the valued trials than baseline trials.

We also analysed the cue data from the selective satiation sessions in a similar manner. First, we again compared all forced trials (food versus sucrose solution) either in food devaluation or sucrose solution devaluation sessions. We then examined dopamine release following presentation of food or sucrose solution cues in a valued or devalued state and contrasted it with dopamine release to that cue in the immediately preceding Baseline session (i.e., if food was devalued in Selective Satiation session B for a particular rat, the Baseline B data was used for comparison whereas if it was devalued in Selective Satiation session A, Baseline A data was used). Once again, the average dopamine levels in the 5s post-cue period on forced trials were taken for each of 5 equally sized bins and analysed. In particular, we focused on changes observed in the first 2-block bin (1st 8 forced trials for each reinforcer).

Finally, we examined the cue data just from the first food and sucrose solution trial in each session before the animals have experienced each reinforcer in the session. If the animal made an error or did not respond on one of these trials, we instead took the first trial where a response was correctly made. We not only extracted the average dopamine signals in a 5s window after cue onset but also in a 2s window after lever extension, during which animals made a lever press response. Note that as there was a 2s delay between the response and reward delivery, none of these signals were contaminated by post-reward changes in dopamine.

Histology

At the end of the experiment, the rats were deeply anaesthetized with sodium pentobarbitone (200mg/kg) and electrolytic microlesions were made at the

electrode locations before they transcardially perfused with saline followed by a 10% formol saline solution. Brains were extracted and placed into a formol saline solution. Subsequently, the brains were placed in a sucrose/formalin solution for 24 hours, before being frozen and sectioned in 50 µm coronal slices using a cryostat. Sections were stained with cresyl violet and electrode locations were verified by two experimenters to confirm the electrode locations (Fig.S1).

Supplementary Figures

mm from Bregma

+ 2.52

+ 2.16

+1.8

+1.68

+1.08

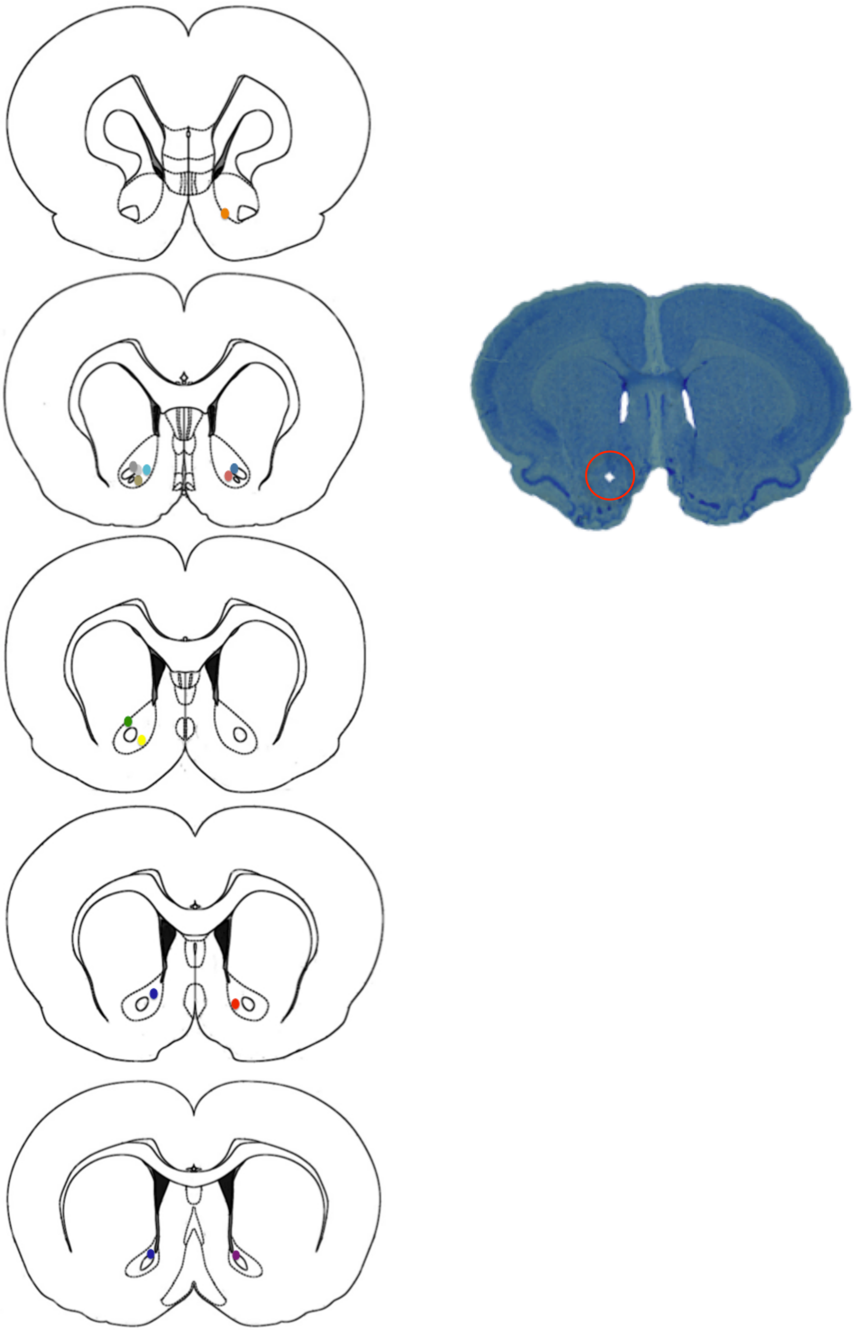

Figure S1. Representation of Recording Sites, Related to the FCV Data in the Results. Schematic, along with an example photomicrograph, of the recording locations in the nucleus accumbens core. The blue dot on the schematic shows the location of the electrode indicated by the red circle on the photomicrograph. The numbers next to each section indicate distance in mm anterior to bregma. Adapted from the atlas of Paxinos and Watson (2005).

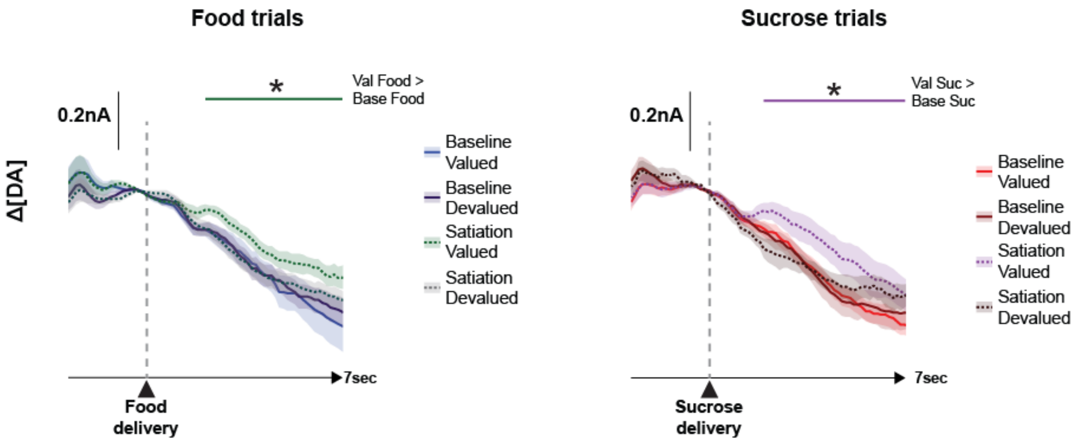

Figure S2. Comparison Between Dopamine Release in Baseline and Devaluation Sessions, Related to Figure 2. Dopamine release time-locked to reward delivery for expected food (left panel) or sucrose solution (right panel) in either the Baseline or Devaluation session. The data are divided up based on which reinforcer type rats had free access to before the Devaluation session: the represented reward ("Satiation Devalued") or the other reward ("Satiation Valued"). Baseline data are from the immediately preceding Baseline session. Therefore, if Food was devalued in Session 2 and Sucrose Solution in Session 4, Baseline A data would be "Baseline Devalued" or Baseline B data would be "Baseline Valued" for Food trials and vice versa for Sucrose Solution trials.
